# Improving radiotherapy in immunosuppressive microenvironments by targeting complement receptor C5aR1

**DOI:** 10.1172/JCI168277

**Published:** 2023-12-01

**Authors:** Callum Beach, David MacLean, Dominika Majorova, Stavros Melemenidis, Dhanya K. Nambiar, Ryan K. Kim, Gabriel N. Valbuena, Silvia Guglietta, Carsten Krieg, Mahnaz Darvish-Damavandi, Tatsuya Suwa, Alistair Easton, Lily V.S. Hillson, Ashley K. McCulloch, Ross K. McMahon, Kathryn Pennel, Joanne Edwards, Sean M. O’Cathail, Campbell S. Roxburgh, Enric Domingo, Eui Jung Moon, Dadi Jiang, Yanyan Jiang, Qingyang Zhang, Albert C. Koong, Trent M. Woodruff, Edward E. Graves, Tim Maughan, Simon J.A. Buczacki, Manuel Stucki, Quynh-Thu Le, Simon J. Leedham, Amato J. Giaccia, Monica M. Olcina

**Affiliations:** 1 Department of Oncology, University of Oxford, Oxford, United Kingdom.; 2 Department of Radiation Oncology, Stanford University, Stanford, California, USA.; 3 Wellcome Centre for Human Genetics, University of Oxford, Oxford, United Kingdom.; 4 Department of Regenerative Medicine and Cell Biology,; 5 Hollings Cancer Center, and; 6 Department of Pathology and Laboratory Medicine, Medical University of South Carolina, Charleston, South Carolina, USA.; 7 Nuffield Department of Surgical Sciences, University of Oxford, Oxford, United Kingdom.; 8 School of Cancer Sciences, University of Glasgow, Glasgow, United Kingdom.; 9 The University of Texas MD Anderson Cancer Center, Houston, Texas, USA.; 10 School of Biomedical Sciences, Faculty of Medicine, The University of Queensland, Brisbane, Queensland, Australia.; 11 Department of Gynecology, University of Zurich, Schlieren, Switzerland.

**Keywords:** Cell Biology, Oncology, Cancer, Complement, Radiation therapy

## Abstract

An immunosuppressive microenvironment causes poor tumor T cell infiltration and is associated with reduced patient overall survival in colorectal cancer. How to improve treatment responses in these tumors is still a challenge. Using an integrated screening approach to identify cancer-specific vulnerabilities, we identified complement receptor C5aR1 as a druggable target, which when inhibited improved radiotherapy, even in tumors displaying immunosuppressive features and poor CD8^+^ T cell infiltration. While C5aR1 is well-known for its role in the immune compartment, we found that C5aR1 is also robustly expressed on malignant epithelial cells, highlighting potential tumor cell–specific functions. C5aR1 targeting resulted in increased NF-κB–dependent apoptosis specifically in tumors and not normal tissues, indicating that, in malignant cells, C5aR1 primarily regulated cell fate. Collectively, these data revealed that increased complement gene expression is part of the stress response mounted by irradiated tumors and that targeting C5aR1 could improve radiotherapy, even in tumors displaying immunosuppressive features.

## Introduction

The composition of the tumor microenvironment (TME) affects treatment responses in cancer (1–3). Immunosuppressive TME features, which act as a barrier to extensive CD8^+^ T cell infiltration typically characterize immune cold tumors, which are associated with poor prognosis (4–6). Indeed, low density of total T lymphocytes (CD3^+^) at the center or invasive margins of tumors is associated with reduced overall survival in colorectal cancer (CRC) (5). Improving treatment responses for those patients with poor tumor lymphocyte infiltration remains a challenge.

Approximately one-third of all CRCs arise in the rectum. Locally advanced rectal cancers are typically treated with neoadjuvant chemoradiotherapy (nCRT) prior to surgery. Unfortunately, despite nCRT leading to complete pathological response in 15%–20% of these patients, 75%–80% will fail to achieve complete responses (7, 8). There is therefore a need to further improve responses in a significant portion of patients receiving nCRT (9–11). Identifying targets that modulate radiosensitivity, particularly in tumors displaying immunosuppressive features, could improve treatment outcomes for the most resistant tumors.

High expression of complement system components is part of the inflammatory environment of colon and rectal tumors displaying the worst survival outcomes (4, 12–14). The complement system is an ancient component of innate immunity, and both canonical and noncanonical functions are increasingly being recognized as important for infection control, autoimmunity, and cancer (15–19). In the context of cancer treatment, complement proteins can be expressed and may function independently of their role in the inflammatory environment; however, this remains to be fully understood.

In this study, we found that, in murine models that recapitulate an immunosuppressive TME, the complement system was the first immune response pathway to be upregulated at early time points following irradiation (RT). Importantly, an enrichment in complement signatures following CRT was also observed in longitudinal biopsies from patients with rectal adenocarcinoma. Through an integrated screening approach, we identified complement receptor C5aR1 as a druggable target, which inhibited radiation-induced cell death/apoptosis through regulation of tumor cell fate. Interestingly, these effects were not observed in untransformed intestinal organoids or normal intestinal tissues in vivo. Consequently, targeting C5aR1 with a clinical grade and orally active C5aR1 antagonist, PMX205, resulted in improved tumor radiation responses in vivo. Importantly, PMX205 improved response in several murine models, including those displaying high radiation-induced complement expression and immunosuppressive features associated with CD8^+^ T cell exclusion.

## Results

### Identification of radiation-responsive targets in immunosuppressive tumors.

**Figure 1:**
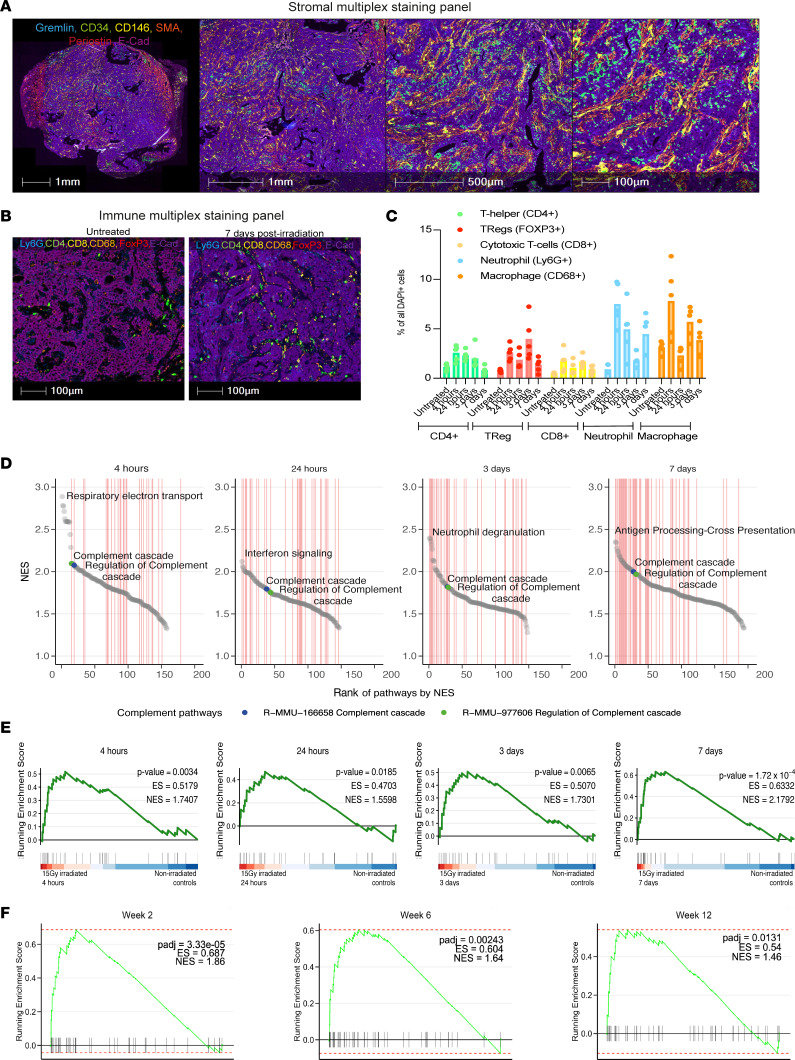
Identification of radiation-responsive targets in immunosuppressive tumors. (**A**) Representative images of *villin*Cre^ER^; *Apc*^fl/fl^; *Kras*^G12D/+^; *Trp53*^fl/fl^ *TgfbrI*^fl/fl^ (AKPT) colorectal tumor organoids grown subcutaneously. Multiplex staining of epithelial and stromal cells is shown in whole tumor (left; scale bar: 1 mm) and zoomed in regions (scale bar: 1 mm, 500 μm, and 100 μm [left to right]). (**B**) Representative images of AKPT colorectal tumor organoids grown subcutaneously and treated with either 0 Gy (left) or 15 Gy (right) (scale bar: 100 μm). Multiplex staining of epithelial and immune cells is shown. (**C**) Machine learning–based quantification of immune cell infiltration in AKPT tumors following multiplex staining at different time points following RT. *n* = 5 per group. (**D**) Ranked normalized enrichment scores (NES) are shown below the network graphs for the significant positively enriched pathways. The complement pathways appear among the top most enriched pathways at each time point after 15 Gy radiation treatment. The top enriched pathway for each plot is also shown for comparison. Ranks of pathways annotated as immune system pathways in Reactome are denoted by the vertical lines in red. *n* = 5 (control, *n* = 9). (**E**) Pairwise gene set enrichment analysis comparing irradiated AKPT tumors at 4 hours, 24 hours, 3 days, or 7 days after 15 Gy compared with unirradiated controls. *P* values, enrichment scores (ES), and NES scores are also provided. Complement gene signatures (described in ref. 17) are shown. *n* = 5 (control, *n* = 9). (**F**) Pairwise gene set enrichment analysis comparing baseline samples to samples collected 2 weeks, 6 weeks, or 12 weeks after starting RT in longitudinal biopsies from patients with rectal adenocarcinoma. *P* values, ES, and NES scores are also provided. Complement gene signatures (described in ref. 17) are shown.

When grown subcutaneously, tumor organoids originally derived from *villin*Cre^ER^; *Apc*^fl/fl^; *Kras*^G12D/+^; *Trp53*^fl/fl^ *TgfbrI*^fl/fl^ (AKPT) mice displayed TME features resembling those of CRC samples from patients that typically have poor outcomes (Figure 1, A–C). These features include stromal-rich regions with high numbers of fibroblasts and macrophages but relatively few CD8^+^ T cells (Figure 1, A–C). Interestingly, we found that although RT was able to moderately enhance infiltration of Tregs, macrophages, neutrophils, and CD4^+^ and CD8^+^ T cells into these tumors, such infiltration was limited to stromal regions and did not increase numbers of intraepithelial immune cells (Figure 1, A–C, and Supplemental Figure 1, A–G; supplemental material available online with this article; https://doi.org/10.1172/JCI168277DS1). To identify radiation-responsive pathways in the immunosuppressive microenvironment of these tumors, we performed RNA-Seq analysis. Network analysis of differentially expressed pathways following RT indicated that the complement cascade was significantly upregulated and, in fact, was the top-ranked pathway, annotated as an “immune system pathway,” in Reactome at early time points after RT (Figure 1, D and E; Supplemental Figure 1H; and Supplemental Table 1). Examples of members across all the main complement functional categories were induced following RT, with most individual genes showing transient enhanced expression (Supplemental Figure 1, I–M). Analysis of enriched pathways in rectal adenocarcinoma biopsies taken at baseline, 2, 6, or 12 weeks during/following nCRT indicated that complement system genes were also significantly upregulated (compared with baseline, Figure 1F and Supplemental Figure 1N). As an example, analysis of the top 50 enriched pathways at week 6 (compared with baseline) indicated that the complement hallmark pathway was ranked fourth. Overall, these data indicate that complement gene expression was induced following RT in both a murine tumor model of an immunosuppressive TME and in patients with rectal cancer undergoing nCRT.

### C5aR1 is a radiation-responsive druggable target.

**Figure 2:**
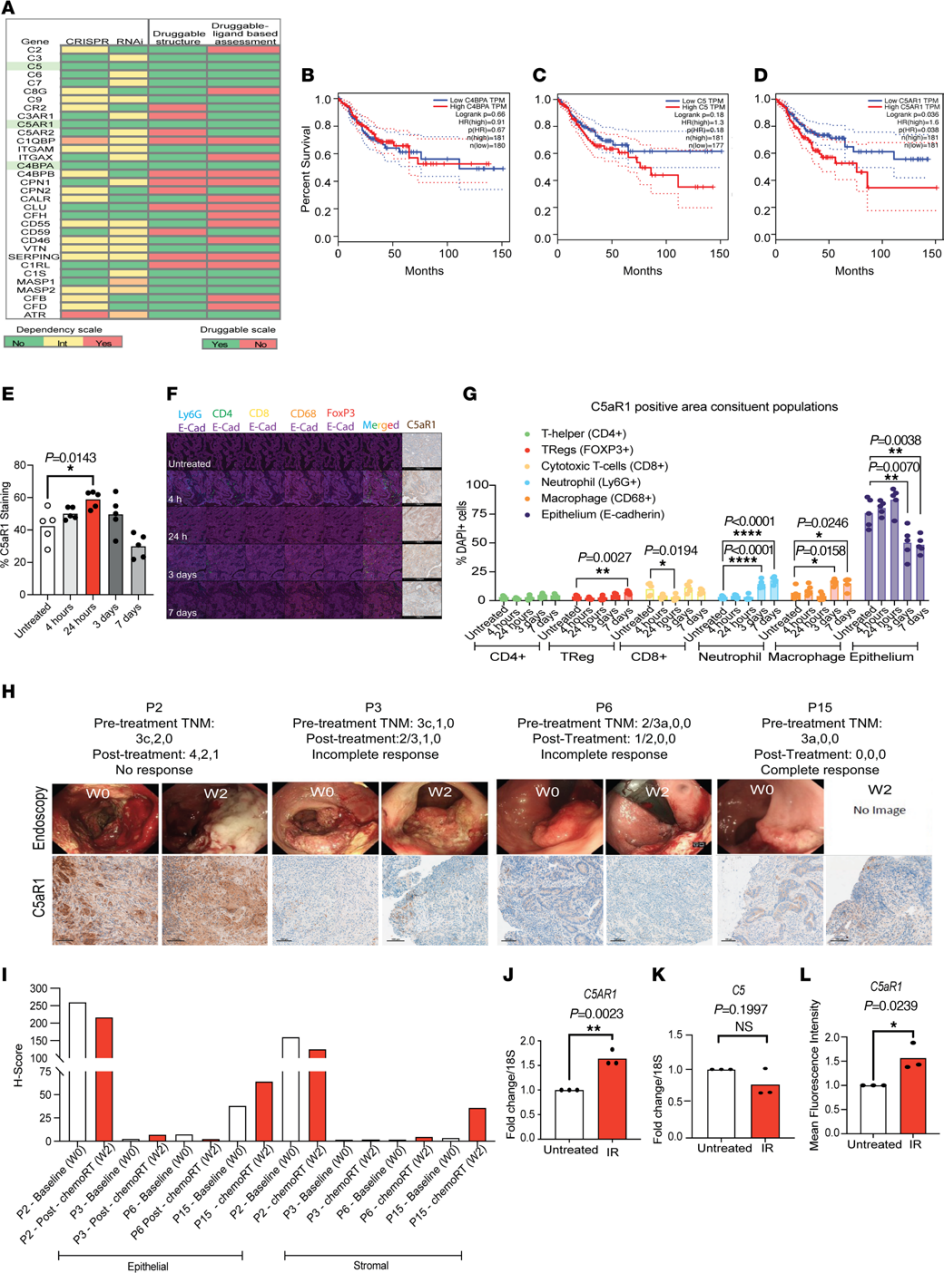
C5aR1 is a radiation-responsive druggable target. (**A**) Essential genes are shown in red. Nonessential genes are shown in green. Yellow/Orange indicates intermediate dependence or essentiality. For target tractability, green corresponds with druggable structure = Yes and druggable by ligand-based assessment = Yes. Red corresponds to druggable structure = No and druggable by ligand-based assessment = No. (**B**) Kaplan-Meier (KM) curve for disease-free survival (dfs) of TCGA patients with CRC with high (red) or low (blue) C4BPA mRNA expression. For all KM curves, group cutoff = median (http://gepia.cancer-pku.cn). (**C**) KM curve for dfs of TCGA patients with CRC with high (red) or low (blue) C5 mRNA expression. (**D**) KM curve for dfs of TCGA CRC with high (red) or low (blue) C5AR1 mRNA expression. (**E**) Quantification of C5aR1 immunohistochemistry staining in AKPT tumors. *P < 0.05, ordinary 1-way ANOVA, Dunnett’s multiple comparisons. All other comparisons relative to untreated were not significant. *n* = 5. (**F**) Representative images of multiplex and C5aR1 IHC staining in AKPT tumors (original magnification, ×40) Scale bar: 500 µm. (**G**) Proximity-based machine learning quantification of the percentage of C5aR1 staining in the epithelium and stroma of AKPT tumors. *P < 0.05, **P < 0.01, ****P < 0.0001, ordinary 1-way ANOVA with Tukey’s multiple comparisons. All other comparisons relative to untreated were not significant. *n* = 5. (**H**) Endoscopy images and representative examples of C5aR1 staining at baseline (W0) compared with W2 in longitudinal biopsies from patients with rectal adenocarcinoma. W2, week 2 after treatment. Numbers refer to patient number, week after treatment, or TNM stage. Scale bar: 100 mm. (**I**) H-Scores of C5aR1 staining in epithelial and stromal areas of cancerous tissue from rectal adenocarcinoma longitudinal biopsies taken at W0 compared with W2. (**J**) mRNA expression of *C5AR1*/housekeeping in HCT116 cells treated with either 0 or 9 Gy. *n* = 3. **P < 0.01, 2-tailed *t* test. (**K**) mRNA expression of *C5*/housekeeping in HCT116 cells treated as in **J**. *n* = 3. Two-tailed *t* test. (**L**) C5aR1 median fluorescence intensity in HCT116 cells treated with either 0 or 9 Gy. *n* = 3. *P < 0.05, 2-tailed *t* test.

To identify potential targets within the complement cascade that could be therapeutically inhibited, we queried the CanSAR database (https://cansar.ai/; ref. 20). A gene was only considered a hit if it was “druggable” based on structural and ligand-based assessment (Figure 2A and Supplemental Table 2, as shown in green). We also interrogated the DepMap database (https://depmap.org/portal/) (which combines data from CRISPR/Cas9 and RNAi screens in more than 700 cell lines) to identify cancer-derived complement proteins that may have autocrine functions specifically affecting cell fate under stress conditions. We reasoned that looking for nonessential hits would allow the identification of genes providing stress-specific dependencies and, therefore, potential therapeutic targets less likely to mediate toxicity in normal tissues. Following the combined CanSAR and DepMap analysis, we found 3 hits: *C5*, *C5AR1*, and *C4BPA* (Figure 2A and Supplemental Table 2). *ATR* was included in the screen as a positive control for essential genes because its deletion is lethal in several cell lines due to its role in replication and the DNA damage response (21, 22). Interestingly, *C1QBP*, a complement gene that was recently shown to play a role in the DNA damage response by modulating DNA resection, was essential in a number of cell lines, further validating our screening approach (23) (Figure 2A and Supplemental Table 2).

*C5* encodes a complement component, which when cleaved will form C5a. C5aR1 is the main signaling receptor for the C5a ligand. *C4BPA* encodes the α chain of complement regulator C4BP (24). There are currently no known pharmacological approaches for targeting C4BPA. To further narrow down which hit would be the best therapeutic target, we assessed the association of C5, C5aR1, and C4BPA mRNA expression with prognostic outcomes and found that only high C5aR1 mRNA expression was associated with significantly poor disease-free survival in CRC (Figure 2, B–D). We confirmed that high C5aR1 mRNA expression was correlated with decreased overall survival in a further independent data set (Supplemental Figure 2A).

In vivo we found that C5aR1 was robustly expressed in AKPT tumors at baseline. A transient induction in C5aR1 expression was also observed after RT (Figure 2E and Supplemental Figure 2, B and C, for negative control C5aR1 staining). We next used multiplex staining and machine learning–based image analysis software to investigate the cellular compartments expressing C5aR1 in greater detail (Figure 2, F and G). Interestingly, we found that at baseline and earlier time points after RT, C5aR1 expression was more prominent in the epithelium, while stromal C5aR1 expression appeared to increase at later time points after RT. Within the immune populations, macrophages and neutrophils were associated with the greatest C5aR1 staining (especially at 3 and 7 days after RT) (Figure 2G). The dominance of stromal C5aR1 expression at these later time points may reflect increased infiltration of C5aR1-expressing immune cells following RT, although this remains to be formally assessed. AKPT tumors harbor stromal infiltration features comparable to those of patients with tumors that could be classified as consensus molecular subtype 4 (CMS4) (4). We therefore asked whether C5aR1 expression might be differentially expressed across subtypes (CMS1–CMS4). Analysis of pretreatment rectal tumor biopsies identified that those classified as CMS4 had the highest RNA levels of C5aR1 compared with the other subtypes (Supplemental Figure 2D). Furthermore, in previously analyzed RNA-Seq of longitudinal biopsies from patients with rectal adenocarcinoma, we noted that C5aR1 expression was significantly increased following treatment (Supplemental Figure 2, E and F). Analysis of C5aR1 staining in these biopsies indicated that C5aR1 expression was higher in malignant, cancerous tissue than in normal, reactive tissue (Figure 2, H and I; Supplemental Figure 2, G and H; and Supplemental Table 3). Within the cancerous tissue, in 3 of 4 patients analyzed, C5aR1 staining remained either high (with >90% of the epithelium staining for C5aR1) or increased following RT (Figure 2, H and I). Heterogeneity in C5aR1 staining was observed with 1 patient showing very high levels of C5aR1 at baseline (P2), 2 patients showing low baseline staining (P3 and P6), and 1 showing intermediate levels of staining (P15). In all patients C5aR1 staining was higher in the epithelium compared with the stroma (Figure 2, H and I, and Supplemental Figure 2, G and H).

To directly assess whether radiation could impact tumor cell–intrinsic expression of C5aR1 or C5, we turned to an in vitro system. Following RT of mouse and human CRC cells, we found a modest but reproducible increase in C5aR1 (but not C5) across cell lines (Figure 2, J–L, and Supplemental Figure 2, I–M). We also noted that GFP-tagged C5aR1 mostly colocalized with phalloidin-labeled cytoskeletal actin filaments, indicating that C5aR1 is likely present at the plasma membrane (Supplemental Figure 2N).

### C5aR1 regulates tumor cell survival under stress.

**Figure 3:**
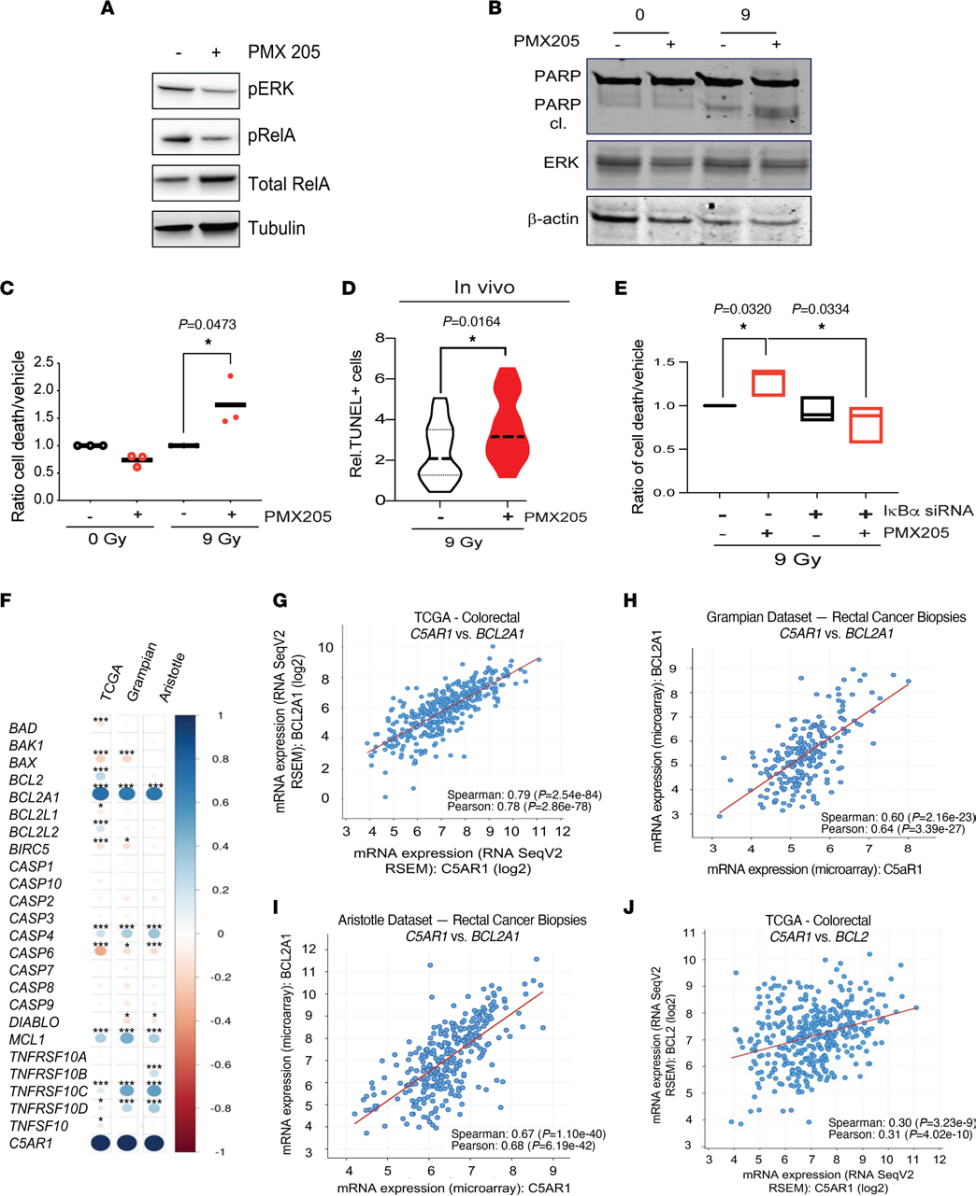
C5aR1 regulates tumor cell survival under stress. (**A**) HCT116 cells were treated with either vehicle or PMX205 (10 mg/mL) for 48 hours. Western blotting was carried with the antibodies indicated. (**B**) HCT116 cells were treated with 0 or 9 Gy and either vehicle or PMX205 for 1 hour before RT. Cells were harvested 48 hours after RT. Western blotting was carried with the antibodies indicated. (**C**) The ratio of dead (apoptotic) cells/nonapoptotic cells as a percentage of the whole population relative to vehicle-treated cells. *n* = 3. *P < 0.05, 2-tailed *t* test. (**D**) TUNEL^+^ cells per field of view of sections from HCT116 subcutaneous tumors. Mice were treated with 9 Gy and either vehicle or PMX205 (10 mg/kg) for 3 doses flanking RT. *P < 0.05, 2-tailed *t* test. (**E**) The number of dead(apoptotic)/nonapoptotic cells as a percentage of the whole population for HCT116 cells transfected with either Scr or IκBα siRNA and treated with either vehicle or PMX205 for 1 hour before RT. Cells were harvested 48 hours after RT. Independent fields of view from a representative experiment are shown, *n* = 3. *P < 0.05, 2-tailed *t* test. (**F**) Pearson’s correlation of mRNA expression of pro- and antiapoptotic genes with C5aR1 in TCGA samples from patients with CRC and rectal cancer biopsies from Grampian and Aristotle. *P < 0.05, ***P < 0.001, ****P < 0.0001. All TCGA data were accessed through cBioPortal (http://www.cbioportal.org/). All Grampian and Aristotle data were accessed through SCORT (stratification in colorectal cancer, http://www.cbioportal.org/). (**G**) Correlation of *BCL2A1* and *C5AR1* mRNA expression in TCGA CRC samples. (**H**) Correlation of *BCL2A1* and *C5AR1* mRNA expression in Grampian rectal cancer biopsies. (**I**) Correlation of *BCL2A1* and *C5AR1* mRNA expression in Aristotle rectal cancer biopsies. (**J**) Correlation of *BCL2* and *C5AR1* mRNA expression in TCGA CRC samples.

PMX205 is a selective inhibitor of C5aR1 currently undergoing clinical testing for ALS; it is reportedly well-tolerated (25). We assessed the effects of treating CRC cells with PMX205. As anticipated, given the fact that C5aR1 is a GPCR, we found reduced ERK1/2 and RelA phosphorylation (as a readout for NF-κB signaling) in PMX205-treated human and mouse cells (Figure 3A and Supplemental Figure 3A). However, PMX205 had negligible effects on AKT phosphorylation (at threonine 308) (Supplemental Figure 3, B and C).

Changes in MAPK signaling could impact cell cycle distribution, which, in turn, could impact cellular radiosensitivity. However, we did not note any significant differences in cell cycle profiles or in cell proliferation in cells treated with or without PMX205 with or without RT (Supplemental Figure 3, D–G). We also did not note significant changes in γH2AX levels or 53BP1 foci between treatment groups, suggesting that DNA damage and repair are likely unaffected by PMX205 (Supplemental Figure 3, H and I). Functional annotation analysis of differentially expressed proteins following reverse-phase protein array (RPPA) analysis indicated that the top differentially expressed pathways at the protein level clustered around apoptosis/cell death with negative regulators of apoptosis being repressed following PMX205 treatment and positive regulators of apoptosis being upregulated (Supplemental Figure 3J and Supplemental Tables 4 and 5). Protein phosphorylation and MAPK cascade were also differentially expressed, consistent with reduced GPCR activity downstream of C5aR1 inhibition and our Western blotting data (Figure 3A). In line with the RPPA data, PMX205 treatment or C5aR1 depletion resulted in increased apoptosis in tumor cells following RT as well as 5-FU and Oxaliplatin treatment (Figure 3, B and C, and Supplemental Figure 3, K–P). Targeting C5aR1 did not result in increased apoptosis in the absence of RT, in agreement with a stress-specific role in modulating cell death. Supporting this, in HCT116 xenografts, we observed increased apoptosis in PMX205- and RT-treated tumors (Figure 3D).

To investigate whether apoptosis of PMX205-treated tumor cells occurred downstream of attenuated GPCR-associated signaling, we first depleted NF-κB inhibitor, IκBα, as a means of interrogating the NF-κB dependence of the effects observed. If PMX205-mediated apoptosis was occurring in an NF-κB–dependent manner, IκBα depletion would be expected to result in decreased apoptosis in PMX205-treated cells. We indeed observed that, following RT, IκBα depletion attenuated the apoptotic response (Figure 3E and Supplemental Figure 3, Q and R). IκBα depletion did not have a dramatic effect on apoptosis levels in the vehicle- and RT-treated cells. We hypothesize this is because IκBα levels are reduced by DNA-damaging agents, and NF-κB signaling is already high in these cells. We also depleted RelA and found that, while RelA depletion increased apoptosis levels in RT- and vehicle-treated cells (as expected), there was no further increase in apoptosis in RT- and PMX205-treated cells (presumably because these cells already have reduced “active” RelA) (Supplemental Figure 3, S and T). Furthermore, interrogation of The Cancer Genome Atlas (TCGA) patient data sets in which high C5aR1 mRNA expression was associated with poor outcome identified that C5aR1 expression was positively and significantly correlated with prosurvival/antiapoptosis genes, including NF-κB target genes in the BCL2 family (Figure 3, F–J, and Supplemental Table 6). We confirmed these associations in two further independent patient data sets, including biopsies from patients with rectal cancer collected prior to radiotherapy (Figure 3, G–J). Treatment of CRC cells with PMX205 also resulted in reduced mRNA expression of BCL2 (Supplemental Figure 3U).

We next assessed the effects of ERK inhibition on PMX205-induced apoptosis by treating CRC cells with PMX205 and ERK inhibitor selumetinib. As expected, PMX205 and selumetinib alone resulted in enhanced apoptosis following RT, with PMX205 displaying the most significant effects (Supplemental Figure 3, V and W). A moderate (yet not significant) increase in apoptosis was also observed when both compounds were combined (Supplemental Figure 3, V and W). These data suggest that although attenuated ERK may contribute to apoptosis following PMX205 treatment, it is unlikely to be a main driver of the apoptotic effect observed. Together, these data suggest that C5aR1 mediates tumor cell prosurvival signaling, with NF-κB acting as a key regulator of this response. In support of this conclusion, increased cell death following PMX205 treatment occurs downstream of attenuated NF-κB signaling.

### C5aR1 deficiency does not result in increased apoptosis in healthy intestinal epithelium.

**Figure 4:**
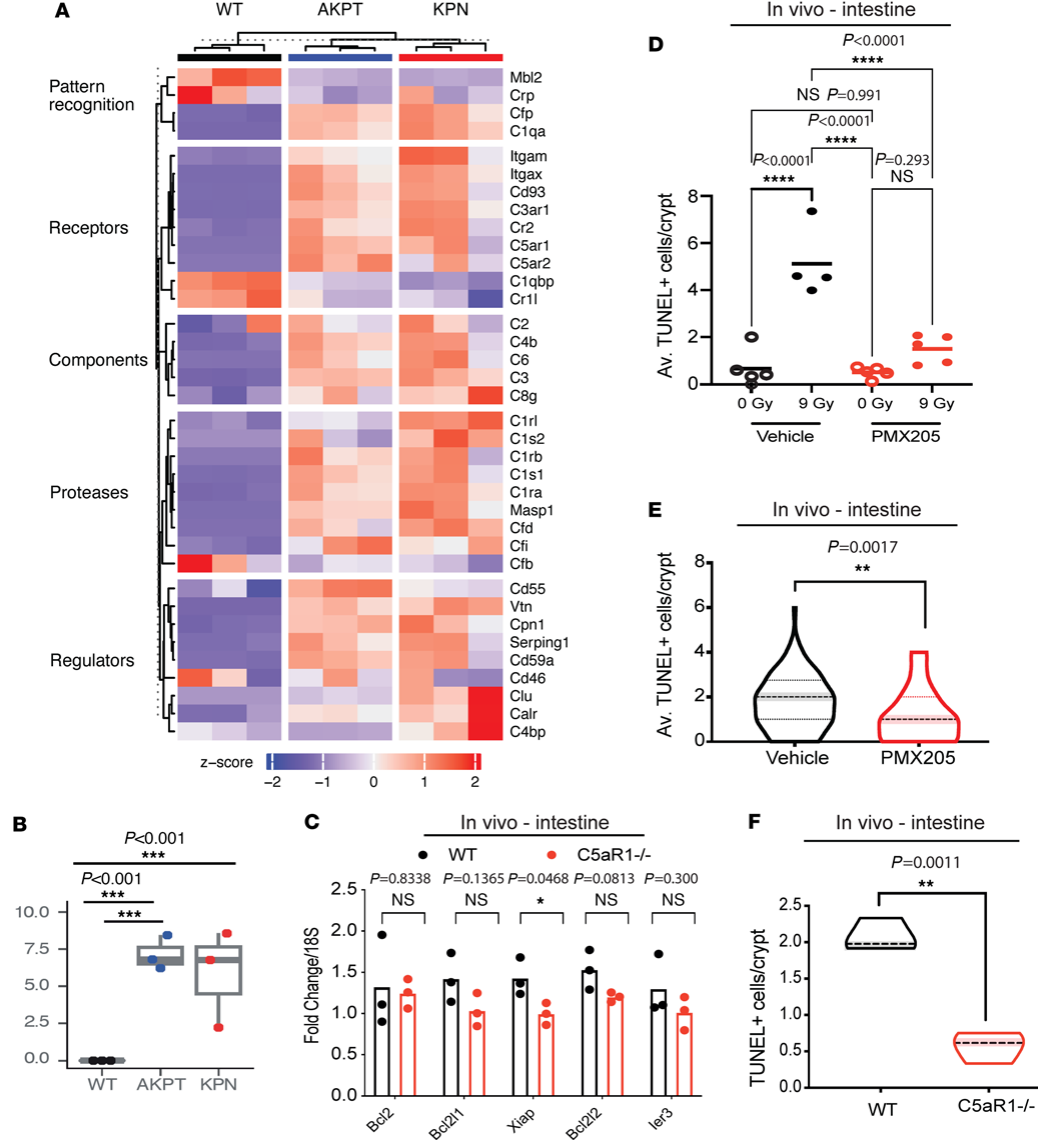
C5aR1 deficiency does not result in increased apoptosis in healthy intestinal epithelium. (**A**) Heatmap of complement genes differentially expressed by RNA-Seq in WT, AKPT, or KPN organoids grown in vitro. Upregulation is shown in red, as per *Z*-score indicated below. Data for 3 independent samples is shown from data deposited at the ArrayExpress database under accession number E-MTAB-11769 (46). (**B**) C5aR1 expression (CPM) assessed by RNA-Seq in WT, AKPT, or KPN organoids grown in vitro as in **A**. ***P < 0.001, empirical Bayes moderated t-statistic test. (**C**) mRNA expression of *Bcl2*, *Bcl2l1*, *Bcl2l2*, *Ier3*, and *Xiap* in WT or C5aR1^–/–^ mice treated with 9 Gy. Points indicate individual mice per group. *n* = 3. *P = 0.0468, unpaired 2-tailed *t* test. (**D**) The average TUNEL^+^ cells/crypt of BALBc/J mice treated with either 0 or 9 Gy and either vehicle or PMX205. Intestines were harvested 72 hours after RT. *n* = 4/5 mice per group. ****P < 0.0001, by ordinary 1-way ANOVA with Tukey’s multiple comparisons. (**E**) The average TUNEL^+^ cells/crypt of C57BL/6 mice treated with 9 Gy and either vehicle or PMX205. Intestines were harvested 72 hours after RT. *n* = 3 mice per group. **P < 0.01, 2-tailed *t* test. (**F**) The average number of TUNEL^+^ cells of WT or C5aR1^–/–^ mice treated with 9 Gy. Intestines were harvested 48 hours after RT. *n* = 3 mice per group. *P < 0.05, 2-tailed *t* test.

To assess whether the cell-intrinsic effects of complement were specific to malignant cells, we first considered if organoids derived from different genotypes expressed complement genes when grown in vitro (and therefore in the absence of systemic complement or TME-derived complement). Interestingly, following RNA-Seq, we found that complement genes were significantly differentially expressed in both AKPT tumor organoids as well as organoids derived from *villin*Cre^ER^; *Kras*^G12D/+^; *Trp53*^fl/fl^ Rosa26^N1icd/+^ (KPN) mice (compared with untransformed WT organoids). Over 74% of the complement genes queried were expressed at significantly higher levels in the AKPT organoids compared with the WT organoids (including C5aR1), suggesting that increased cell-intrinsic complement expression is a malignant cell-associated phenomenon (Figure 4, A and B).

To investigate whether targeting C5aR1 might alter prosurvival signaling in healthy tissues, we assessed RelA and ERK phosphorylation in untransformed intestinal organoids. We noted that, while ERK phosphorylation was reduced following PMX205 treatment, RelA phosphorylation was not affected (Supplemental Figure 4A). We also performed RNA-Seq in intestinal untransformed organoids treated with or without PMX205 and with or without RT. Following gene ontology analysis, as expected, genes within the GPCR activity pathway were among those differentially expressed (downregulated) in untransformed organoids treated with PMX205 (Supplemental Figure 4, B and C). In line with the lack of RelA phosphorylation changes observed by Western blotting, we noted that transcriptional NF-κB target genes were not differentially expressed in PMX205- and RT-treated versus RT-treated organoids (Supplemental Figure 4D). Similarly, in vivo, small intestines did not show significant transcriptional changes in antiapoptotic target genes in the BCL2 family with deletion of C5aR1 (following RT) (Figure 4C). These data suggest that changes in NF-κB antiapoptotic signaling downstream of C5aR1 do not occur in the untransformed intestinal epithelium. In line with these signaling changes, we did not observe an increase in apoptosis in small intestines following PMX205 treatment or C5aR1 loss (Figure 4, D–F). In fact, small intestinal crypts in vivo had significantly reduced apoptosis following PMX205 treatment or C5aR1 loss following total abdominal RT (Figure 4, D–F). Together, these data indicate that C5aR1 attenuates stress-induced apoptosis in malignant but not nontransformed epithelial cells.

### C5aR1 inhibition improves tumor radiation response.

**Figure 5:**
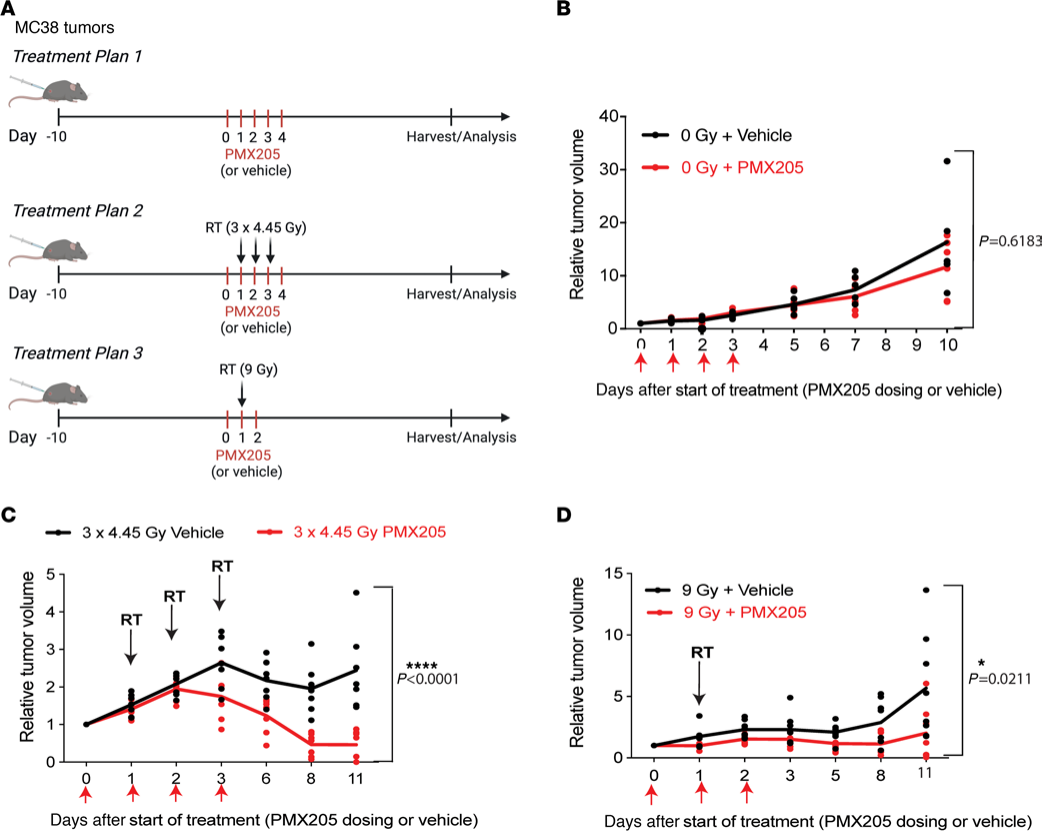
C5aR1 inhibition improves tumor radiation response. (**A**) Schematic representation of the treatment schemes followed. Created with BioRender.com. (**B**) Relative tumor growth curves are shown for MC38 subcutaneous tumors treated with either vehicle or PMX205 treatment for 3 doses (on day 0, 1, and 2). *P* = 0.6183 by repeated measures 2-way ANOVA (with Geisser-Greanhouse correction). *n* = 7 for PMX205; *n* = 6 for vehicle. (**C**) Relative tumor growth curves are shown for MC38 subcutaneous tumors treated with 3 × 4.45 Gy and either vehicle or PMX205 treatment for 3 doses flanking RT. Individual points represent individual mice per group. ****P < 0.0001 by repeated measures 2-way ANOVA (with Geisser-Greanhouse correction); *P* = 0.0073 for day 3; *P* = 0.0048 for day 6; *P* = <0.0001 for day 8 and 11 with Šídák’s multiple comparison test. *n* = 7 for both groups. (**D**) Relative tumor growth curves are shown for MC38 subcutaneous tumors treated with single-dose 9 Gy and either vehicle or PMX205 treatment for 3 doses flanking RT. Individual points represent individual mice per group. *P* = 0.0211 by repeated measures 2-way ANOVA (with Geisser-Greanhouse correction); *P* = 0.0001 for day 11 with Šídák’s multiple comparison test. *n* = 7 for PMX205; *n* = 8 for vehicle.

To assess whether targeting C5aR1 could improve radiation responses in vivo, we treated MC38 subcutaneous tumors with PMX205 and no RT, fractionated RT (3 × 4.45 Gy), or single-dose RT (9 Gy) (Figure 5A and Supplemental Figure 5A). PMX205 treatment in the absence of RT did not have significant effects on tumor response (Figure 5B and Supplemental Figure 5B). PMX205 treatment also had no negative effects on mouse weight (Supplemental Figure 5A). However, following treatment with either single-dose or equivalent fractionation regimens (equivalent assuming an α/β ratio of 5.06) (26) PMX205 treatment significantly improved radiation response (Figure 5, C and D, and Supplemental Figure 5, C and D).

### Targeting C5aR1 does not increase the percentage of CD8^+^ T cells in the tumor following RT.

**Figure 6:**
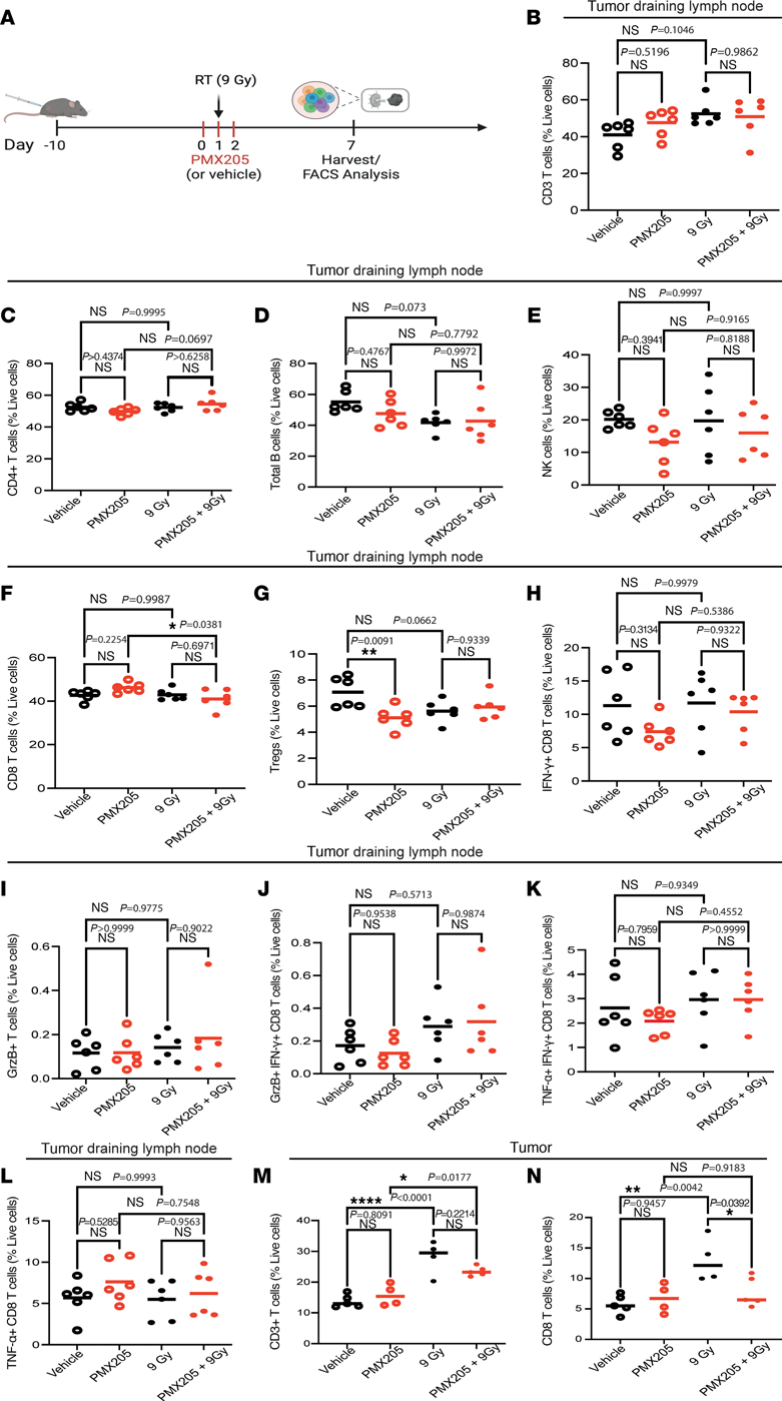
Targeting C5aR1 does not increase the percentage of CD8^+^ T cells in the tumor following RT. (**A**) Schematic representation of the experimental design for **B**–**N**. Created with BioRender.com. (**B**) CD3^+^ T cells in tumor draining lymph nodes following the dosing scheme shown in **A**. Tumors were harvested 7 days after RT. Ordinary 1-way ANOVA with Tukey’s multiple comparisons test. *n* = 5. (**C**) CD4^+^ T cells in tumor draining lymph nodes following the dosing scheme, harvesting schedule, and statistical analysis shown in **B.** *n* = 5. (**D**) Total B cells in tumor draining lymph nodes following the dosing scheme, harvesting schedule, and statistical analysis shown in **B**. *n* = 5. (**E**) Total NK cells in tumor draining lymph nodes following the dosing scheme, harvesting schedule, and statistical analysis shown in **B**. *n* = 5. (**F**) CD8^+^ T cells in tumor draining lymph nodes following the dosing scheme, harvesting schedule, and statistical analysis shown in **B**. *n* = 5. (**G**) Tregs in tumor draining lymph nodes following the dosing scheme, harvesting schedule, and statistical analysis shown in **B**. *n* = 5. (**H**) IFN-γ^+^ CD8 T cells in tumor draining lymph nodes following the dosing scheme, harvesting schedule, and statistical analysis shown in **B**. *n* = 5. (**I**) GrzB^+^CD8 T cells in tumor draining lymph nodes following the dosing scheme, harvesting schedule, and statistical analysis shown in **B**. *n* = 5. (**J**) GrzB^+^IFN-γ^+^ CD8 T cells in tumor draining lymph nodes following the dosing scheme, harvesting schedule, and statistical analysis shown in **B**. *n* = 5. (**K**) TNF-α^+^ IFN-γ^+^ CD8 T cells in tumor draining lymph nodes following the dosing scheme, harvesting schedule, and statistical analysis shown in **B**. *n* = 5. (**L**) TNF-α^+^ CD8 T cells in tumor draining lymph nodes following the dosing scheme, harvesting schedule, and statistical analysis shown in **B**. *n* = 5. (**M**) CD3^+^ T cells in tumors following the dosing scheme, harvesting schedule, and statistical analysis shown in **B**. *n* = 5. (**N**) CD8^+^ T cells in tumors following the dosing scheme, harvesting schedule, and statistical analysis shown in **B**. *n* = 5.

Targeting C5aR1 as a means of reinvigorating antitumor CD8^+^ T cell responses has undergone clinical testing (27). Because we had observed stromal C5aR1 expression in irradiated tumors, we next investigated immune infiltration changes in tumor draining lymph nodes and tumors following PMX205 and RT (Figure 6A). Interestingly, no significant changes in CD3, CD4, NK, or B cells were found in the tumor draining lymph nodes across treatment groups (Figure 6, B–E, and Supplemental Figure 6A). Mice treated with PMX205 alone, however, displayed a higher percentage of CD8^+^ T cells compared with those treated with PMX205 and RT (Figure 6F). Mice treated with PMX205 alone also had a reduced percentage of Tregs compared with vehicle-treated mice (Figure 6G). However, these changes did not correlate with altered functionality/effector functions of CD8^+^ T cells, which showed comparable expression of IFN-γ, GrzB, and TNF-α across all treatment groups (Figure 6, H–L). In the tumor, we found that, although RT, as expected, significantly increased levels of CD3^+^ and CD8^+^ T cells, treatment with PMX205 did not further increase the percentage of these cells (Figure 6, M and N, and Supplemental Figure 6B). In fact, the percentage of CD8^+^ T cells was significantly reduced in tumors after PMX205 and RT treatment when compared with RT vehicle-treated mice (Figure 6N). This was surprising, given the improved tumor regression previously observed in these models (Figure 5D). No significant changes in NK cells or either monocytic or granulocytic myeloid derived suppressor cells were observed across treatments (Supplemental Figure 6, C–F). These data indicated that improved radiation responses following PMX205 treatment can occur despite reduced tumor CD8^+^ T cell infiltration changes.

### C5aR1 inhibition can improve radiotherapy in tumors with an immunosuppressive microenvironment.

**Figure 7:**
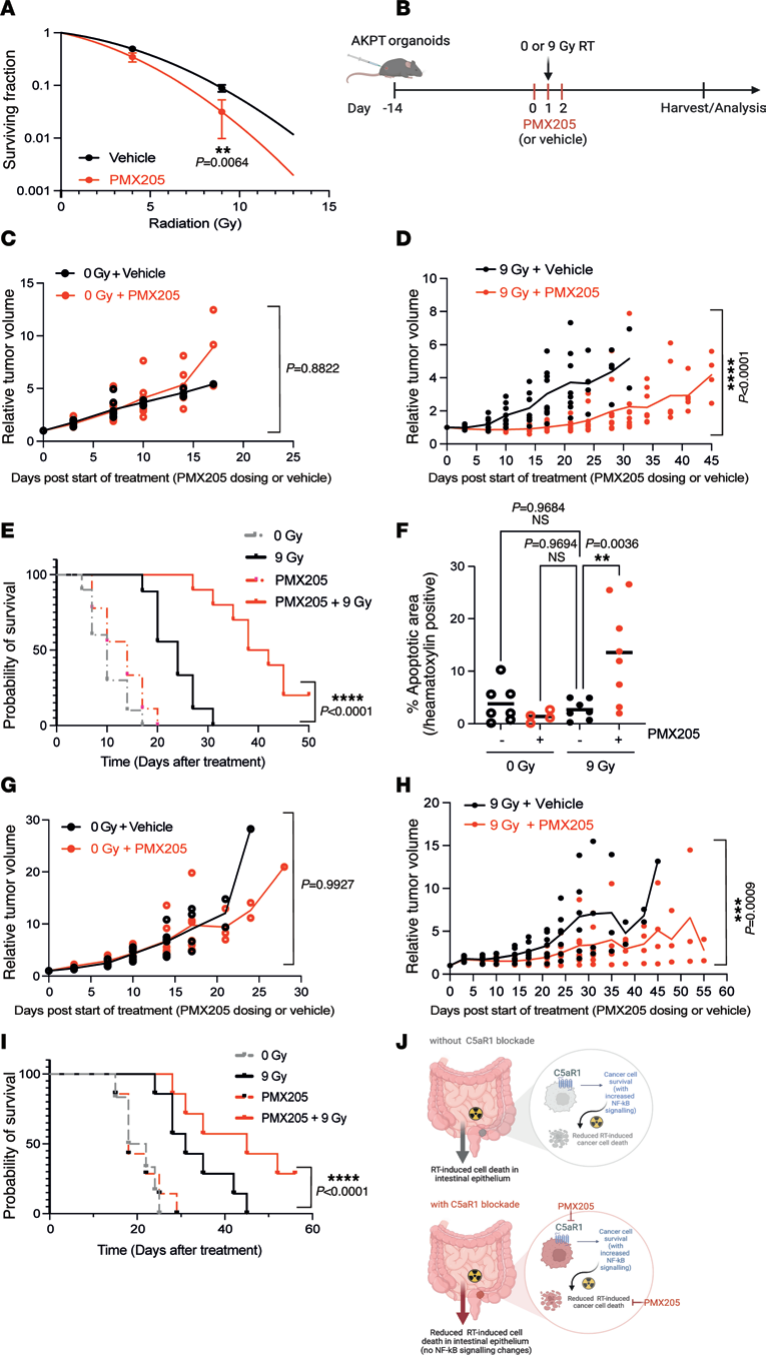
C5aR1 inhibition can improve radiotherapy in tumors with an immunosuppressive microenvironment. (**A**) Curves show counted viable AKPT organoids vs. RT dose following treatment with vehicle or PMX205. **P < 0.01, Welch’s *t* test. Data are shown as the mean. Error bars represent standard deviation. (**B**) Schematic representation of experimental design for **C**–**F**. Created with BioRender.com. (**C**) Tumor growth curves for AKPT organoids grown subcutaneously and treated with 0 Gy and vehicle or PMX205. Comparisons were not significant (*P* = >0.05) by repeated measures 2-way ANOVA (with Geisser-Greanhouse correction). *n* = 7 mice/group. (**D**) Tumor growth curves for AKPT organoids grown subcutaneously and treated with 9 Gy and vehicle or PMX205 flanking RT. ****P < 0.0001 by repeated measures 2-way ANOVA (with Geisser-Greanhouse correction); *P* < 0.05 for day 10, 14, and 17 with Šídák’s comparison test. *n* = 7 mice/group. (**E**) Probability of survival for tumor-bearing mice from **C** and **D**. ****P < 0.0001, log-rank. *n* = 7 mice/group. (**F**) The percentage of apoptotic (TUNEL^+^) area/hematoxylin^+^ area in mice from **C** and **D**. **P < 0.01, ordinary 1-way ANOVA with Dunnett’s multiple comparisons test. *n* = 4–8 mice/group. (**G**) Tumor growth curves for AKPT organoids grown subcutaneously in athymic nude mice and treated with 0 Gy and vehicle or PMX205. Comparisons were not significant (*P* > 0.05) by repeated measures 2-way ANOVA (with Geisser-Greanhouse correction). *n* = 7 mice/group. (**H**) Tumor growth curves for AKPT organoids grown subcutaneously in athymic nude mice and treated with 9 Gy and vehicle or PMX205 flanking RT. ***P < 0.001, repeated measures 2-way ANOVA (with Geisser-Greanhouse correction); ***P < 0.001 for day 24, Šídák’s comparison test. *n* = 7 mice/group. (**I**) Probability of survival for mice from **G** and **H**. ****P < 0.0001, log-rank. *n* = 7/group (*n* = 6 for 0 Gy vehicle). (**J**) Working model: C5aR1 attenuates RT-induced tumor cell death via increased prosurvival signaling (including NF-κB). Upon C5aR1 blockade, tumor cells undergo increased RT-induced cell death, which is not observed in the intestinal epithelium. Created with BioRender.com.

The improved tumor responses observed in the context of reduced tumor CD8^+^ T cell numbers made us question whether PMX205 could be used to improve radiation responses in models displaying low CD8^+^ T cell infiltration. We, therefore, next asked whether PMX205 could improve response in the AKPT model where we had previously observed robust C5aR1 expression and low CD8^+^ T cell tumor infiltration. In support of C5aR1 having cell-intrinsic effects, we observed that, in vitro, PMX205 reduced survival of AKPT organoids upon treatment with increasing RT doses (Figure 7A). We also observed reduced RelA (and Erk) phosphorylation by Western blotting in PMX205-treated AKPT organoids (Supplemental Figure 7A). When these organoids were grown as subcutaneous tumors, PMX205 alone had no significant effect on tumor growth (as previously observed in the MC38 model) (Figure 7, B and C, and Supplemental Figure 7B). However, combination treatment with PMX205 and RT resulted in a significant tumor growth delay, increased apoptosis, and dramatic improvement in tumor-free survival, with 20% of mice having impalpable tumors at the end of the experiment (Figure 7, D–F, and Supplemental Figure 7B). To investigate the T cell dependence of these effects, we repeated the AKPT subcutaneous experiment in athymic nude mice. We noted that improve tumor response and survival was maintained in this model (Figure 7, G–I, and Supplemental Figure 7C). Together, our data indicate that targeting C5aR1 can improve radiation response even in models with low (or absent) tumor CD8^+^ T cell infiltration. Importantly, improved responses are associated with increased tumor cell apoptosis and without concomitant increases in healthy intestinal epithelial cell apoptosis (Figure 7J).

## Discussion

Identifying tumor-promoting components of the TME presents therapeutic opportunities. However, expression of these components can be extremely dynamic and may be governed by selective pressures, such as those posed by treatment-induced stress responses. The effects of these selective pressures on dysregulation of complement components and their evolving functions in the TME is unclear. Here, we report that complement gene expression was induced after RT in murine tumor models that recapitulate features of human tumors displaying the worse outcomes. Importantly, a significant enrichment in complement gene signatures was also found when analyzing biopsies from patients with rectal adenocarcinoma during and after CRT. Among these genes, C5aR1 expression was transiently induced following radiotherapy, likely as a stress response mounted to promote tumor cell survival. Interestingly, in AKPT tumor models, C5aR1 was expressed by both the tumor epithelium and stroma. We found that epithelial expression was more prominent at baseline and early time points after RT, while stromal expression dominated at later time points. Increased C5aR1 stromal expression at later time points may reflect recruitment of C5aR1 expressing immune cells following RT (as indicated in Figure 1C and Supplemental Figure 1, A–G). Interestingly, in patients with rectal cancer, we found that those classified as CMS4 have the highest levels of C5aR1 expression compared with the other subtypes. Analysis of C5aR1 expression in rectal adenocarcinoma biopsies also points to a potential “Goldilocks” effect to C5aR1 expression. In the samples analyzed, very high baseline expression was observed in the patient displaying overt radiation resistance and tumor progression during treatment, low expression was observed in both patients with partial responses, while intermediate epithelial expression (concomitant with increased stromal expression following treatment) was observed in the patient with exquisite radiation sensitivity and a complete response. Future studies with additional samples will be necessary to fully investigate these associations and how to use this information clinically. Together, our data indicate that patients with tumors displaying immunosuppressive features and high C5aR1 expression could represent populations most likely to benefit from C5aR1-targeting therapy.

Previous reports investigating the effects of complement inhibition on radiotherapy response have focused on complement’s role in modulating antitumor immunity, albeit with conflicting results (28, 29). Furthermore, whether targeting complement protein would have the same effects in tumor and normal tissues had, until this study, remained unexplored to our knowledge. Using a combination of RNA-Seq and in silico mining of DepMap, CanSAR, and patient data sets, we identified C5aR1 as a druggable target for enhancing stress-specific cancer cell death. Our data indicate that C5aR1 negatively regulates apoptosis in cancer cells by modulating cell survival pathways, such as NF-κB, and that attenuating such prosurvival signaling can render cancer cells more susceptible to death following RT. Importantly, in the normal intestine, targeting C5aR1 does not result in increased apoptosis, and, in fact, C5aR1 deficiency appears to confer a protective phenotype. Why or how C5aR1 may differentially regulate signaling in normal and malignant cells remains to be elucidated. However, divergent consequences of autocrine complement signaling between cell lines have been previously reported (30). To therapeutically target C5aR1, we used the specific antagonist PMX205, which has FDA and EMA “orphan drug” designation for ALS, allowing accelerated progression to clinical trials (31). This class of cyclic peptides have minimal penetrance into the cell (32) and should therefore primarily impact cell-surface C5aR1. This is important because recent reports indicate that C5aR1 intracellular pools are present in tumor cells where they contribute to tumorigenesis through β-catenin stabilization (33).

We have compared, for the first time to our knowledge, single and equivalent fractionation doses in CRC models to specifically assess the effect of targeting C5aR1. In both settings, we observed that PMX205 improves radiation response, although, interestingly, the effect appeared more pronounced in the fractionated setting. We propose that increased PMX205-mediated cell death is a mechanism that can be exploited to improve responses, even in tumors displaying immunosuppressive features. The importance of modulating antitumor immune host responses following C5aR1 targeting has been highlighted in previous studies, and ourselves and others have observed reduced tumor burden in C5aR1^–/–^ mice (data not shown) (28, 33, 34). The defect in tumor uptake displayed by C5aR1^–/–^ mice complicates the investigation of radiation responses in this model and might have contributed to some of the previous conflicting reports. The reduced tumor CD8^+^ T cell infiltration observed here, however, is in line with reports, indicating that local production of anaphylatoxins C3a and C5a and signaling through their receptors is required for dendritic cell maturation and CD8^+^ T cell activation (28). We acknowledge, however, that more extensive immunophenotyping at different time points following both single and fractionated RT would be required to fully understand the effects of C5aR1 targeting on modulating antitumor immune host responses.

Overall, this study indicates that increased complement gene expression is part of the stress response mounted by irradiated tumors to sustain survival via C5aR1 signaling. These data therefore indicate that, beyond its previously described functions, C5aR1 can also sustain tumor cell survival in a cell-intrinsic manner. Consequently, targeting C5aR1 can improve radiotherapy, even in tumors displaying reduced CD8^+^ T cell infiltration. Importantly, C5aR1’s prosurvival functions appear to be malignant cell specific, because increased apoptosis was not observed in the normal intestinal epithelium following C5aR1 loss. This work is relevant since identifying targets that specifically modulate cancer cell radiosensitivity and can do so even in the absence of robust tumor CD8^+^ T cell infiltration could lead the way to improving treatment outcomes for the most difficult to treat tumors.

## Methods

### Cell lines and treatments.

HCT116 male adult human epithelial CRC cells and HT-29 female adult CRC cells originally purchased from ATCC were used. MC38 murine C57BL6 colon adenocarcinoma cells were provided in-house. Cells were grown in DMEM with 10% FBS, in a standard humidified incubator at 37°C and 5% CO_2_. All cell lines were routinely tested for mycoplasma and found to be negative. Oxaliplatin (Sigma-Aldrich) and 5-FU (Cayman Chemical Company) were used at 40 μM. RT treatment in vitro was carried out using a Gamma Service GSR D1 irradiator containing a Cs137 source. The dose rates of the system, as determined by the supplier, were 1.938 Gy/min and 1.233 Gy/min, depending on the distance from the source.

### AKPT organoid culture.

Organoids were sourced from Eoghan Mulholland (Wellcome Trust Centre Human Genetics, University of Oxford), working within the ACRCelerator: Colorectal Cancer Stratified Medicine Network (A:CCSMN). These were generated from male mice with tumors from liver metastasis formed from a tamoxifen-induced CRC mouse model. Organoids were grown within 35 μL Matrigel and DMEM/F12 media mixture (2:1) with 500 μL media overlaid within each well of a 24-well plate. Overlaid medium was supplemented with epidermal growth factor (EGF) and noggin at a concentration of 100 ng/mL and 50 ng/mL, respectively. Organoids were maintained through passaging every 3 days.

For survival fraction studies, organoids were used 24 hours after passaging and plated into a 24-well plate. Vehicle or PMX205 treatment was added into the surrounding media 1 hour before RT. RT was carried out using an X-ray irradiator with lead shielding, allowing for cumulative doses across a single plate. Organoids were imaged using a 4× objective over 3 days with a JuLi stage Real-Time Cell History recorder (NanoEnTek Inc.). Organoids were manually counted at day 0 and 3, with a surviving fraction subsequently calculated.

### Mouse intestinal organoids.

Establishment and culture of mouse intestinal organoids was carried out as previously described with some modifications (35, 36). Briefly, approximately 2 mm intestinal pieces were rinsed twice with cold PBS and transferred into a 50 mL falcon tube with 30 mL 10% FBS/PBS solution. The tube was shaken forcefully, and the 10% FBS/PBS solution was replaced with 25 mL of HBSS/EDTA solution (without Ca^2+^ and Mg^2+^, Thermo Fisher Scientific). The tube was incubated in 37°C water bath for 10 minutes and shaken forcefully several times every 2–3 minutes. The supernatants containing the isolated crypts were collected, and the procedure was repeated twice. After filtration, centrifugation and washing of isolated crypts, the supernatant was removed and pellets were resuspended in cold Cultrex UltiMatrix Reduced Growth Factor (RGF) BME (Bio-Techne). The BME was then solidified and overlaid with 500 μL complete mouse organoid media (1× DMEM/F-12 [Thermo Fisher Scientific], 1× GlutaMAX [Thermo Fisher Scientific], 10 mM HEPES [Lonza], 10 μg/mL Primocin [InvivoGen], 1× N-2 Supplement [Thermo Fisher Scientific], 1× B-27 Plus Supplement [Gibco], 0.1% BSA, 25% R-spondin-1 conditioned medium [Trevigen], 10 ng/mL mouse EGF [PeproTech], 100 ng/mL mouse noggin [PeproTech], and 3 μM CHIR 99021 [Tocris]) with 10 μM Y-27632. Complete media was subsequently refreshed every two days. Organoids were passaged every 5–10 days by a mechanical approach using Gentle Cell Dissociation Reagent (STEMCELL).

### PMX205 treatment.

For all in vitro experiments, cells were treated with 10 mg/mL PMX205 (Tocris, 5196) dissolved in 20% ethanol/water. Vehicle control in these experiments refers to 20% ethanol/water or water alone. Cells were pretreated for 1 hour before RT.

### Animal studies.

For all studies, animals were randomly divided into groups using a computer-based random order generator. Blinding during data collection and analysis was carried out whenever possible. For Figure 5; Figure 6, M and N; and Supplemental Figure 6, B–F, MC38 cells (5 × 10^5^) were injected subcutaneously into 6- to 8-week-old female C57BL/6 mice (JAX) at a single dorsal site. Animals with growing tumors (average, 80–100 mm^3^) were included in the study and treated with either 9 Gy as a single dose or 3 × 4.45 Gy and either vehicle or PMX205 for the days flanking RT. For RT, mice were anesthetized in a knockdown chamber with a mixture of 3% isoflurane and 100% O_2_ and placed inside the irradiator cabinet on the subject stage. Anesthesia was maintained using 1.5% isoflurane in O_2_ delivered via a nose cone. The X-Rad SmART (Precision X-Ray Inc.) was used. RT was performed using an X-ray energy of 22 5kVp, a current of 13 mA, a power of 3,000 watts, and a beam filter of 0.3 mm Cu, producing a dose rate of approximately 300 cGy/min at the isocenter. Treatment X-ray beams were shaped using a 10 or 15 mm collimator to selectively irradiate the target while sparing adjacent tissue. Pretreatment CT images were acquired, using a beam energy of 40 kVp, a beam filter of 2 mm Al, and a voxel size of 0.2 or 0.1 mm. The open-source RT_Image software package, version 3.13.1, running on IDL version 8.5.1 was used to visualize CT images and perform treatment planning (37).

Subcutaneous tumors were measured with the use of calipers, and volumes were calculated by the ellipsoid estimation method as previously described (38). Mice were euthanized as per Stanford University’s Administrative Panel on Laboratory Animal Care (APLAC) guidelines in a CO_2_ chamber and by cervical dislocation.

For analysis of immune populations in tumor draining lymph nodes, 5 × 10^5^ MC38 cells were injected into the flank of 8-week-old female C57BL/6 mice. When tumors were approximately 50 mm^3^ in volume, the mice were treated via oral gavage, as described in Figure 6. Tumor-bearing mice were anesthetized with ketamine/xylazine (80 mg/Kg/10 mg/Kg), and a dose of 9 Gy was delivered using a Varian TrueBeam 2041 clinical linear accelerator.

For heterotopic AKPT organoid-derived tumor models, female C57BL/6 or athymic nude (Crl:NU(NCr)-*Foxn1^nu^*) mice were purchased from Charles River at 5 to 6 weeks of age. AKPT organoids were suspended in a PBS and Matrigel mixture (1:1) prior to subcutaneous injection. Resulting tumors were monitored once a day, and their volume was determined from the following formula: length × width × height × 0.52 using caliper measurements. The Gulmay Medical RS320 irradiator (300 kV, 10 mA, 1.81 Gy/min) was used for RT. For experiments shown in Figures 1 and 2, animals with growing tumors were included in the study. Once the mean tumor volume across mice was 150–200 mm^3^, mice were randomly divided into groups (as described above). For the experiments shown in Figure 7, animals with growing tumors were included in the study. Once mean tumor volume for all mice reached 100 mm^3^, PMX205/vehicle was delivered by oral gavage once a day for 3 days. Once tumor size reached a length by width 1.2 cm geometric mean mice were euthanized by a Schedule 1 method (in line with the UK Animals [Scientific Procedures] Act 1986 [ASPA]).

For normal tissue studies, animals with stable body weights were included in the study. Total abdominal RT of either C57BL/6 (JAX) (treated with vehicle or PMX205) or WT and C5aR1^–/–^ BALBc/J (JAX) mice was performed on anesthetized animals (with the use of ketamine 100 mg/kg/xylazine 20 mg/kg) and using a 225 kVp cabinet X-ray system filtered with 0.5 mm Cu (at Comparative Medicine Unit). Total abdominal RTs were also occasionally carried out using the X-Rad SmART irradiator with the 20 mm collimator with 2 beams (0 and 180 degrees). Mice were euthanized as per Stanford University APLAC guidelines in a CO_2_ chamber and by cervical dislocation.

For in vivo experiments involving PMX205 treatment, 10 mg/kg PMX205 (Tocris, 5196, or synthesized and purified as previously described, ref. 25) was administered to mice orally. Vehicle in these experiments refers to 20% ethanol/water.

### Cell cycle analysis.

Cells were harvested with trypsin, washed in PBS, and fixed in 1 mL 70% ethanol. Following a 30-minute incubation on ice, cells were washed twice in PBS. Cell pellets were treated with 50 μL PureLink RNase A (Invitrogen, 12091-021, 100 μg/mL) before adding 400 μL propidium iodide solution (Invitrogen, 00-6990-50, 50 μg/mL) in PBS. Following a 10-minute incubation at room temperature, cell cycle was analyzed with CytoFLEX Flow Cytometer (Beckman Coulter Inc.), and data analysis was performed using FlowJo Software (version 10.7.2, Tree Star Inc.).

### Proliferation assays.

HCT116 or MC38 cells were seeded at the indicated concentration. Cells were treated with 10 mg/mL PMX205 (or RNAse-Free water as vehicle). On day 2, cells were counted using a hemocytometer and trypan blue in a 1:1 ratio. On day 3, 10 mg/mL PMX205 (or water) was added to the dishes. On day 4, the cells were counted in the same manner as above. Number of cells were plotted normalized to the seeding density.

### Immunohistochemistry.

FFPE 4 μm sections of C5aR1^–/–^ BALBc/J intestines were dewaxed in Histoclear (10 minutes × 2) followed by rehydration in 100% ethanol (5 minutes × 2), 70% ethanol (5 minutes), and 50% ethanol (5 minutes). Sections were then stained with primary C5aR1 antibody (1:1000, Abcam, catalog ab59390) using the EnVision G2 Doublestain System (Dako) according to the manufacturer’s instructions. The whole section was scanned and analyzed using the Aperio CS scanner and ImageScope analysis software (Aperio Technologies).

### TUNEL assay.

ApopTag (Millipore, S7100) was used to stain 4 μm FFPE small intestinal or AKPT tumor sections. Intestine stained slides were scanned with a NanoZoomer 2.0-RS Digital Slide Scanner (Hamamatsu). For small intestines, the number of TUNEL^+^ cells per crypt (or crypts and villi) was manually counted in at least 10 fields of view per section. Stained slides for AKPT tumors were scanned with a Aperio CS scanner (Aperio Technologies), and images were analyzed with QuPath software. The tumor areas were identified on the basis of the histological structure, and the TUNEL^+^ areas were then normalized to hematoxylin^+^ areas to calculate the percentage of TUNEL^+^ cancer cells.

Apoptosis assessment by morphology was carried out as previously described (39, 40) and Mendeley Data, https://data.mendeley.com).

### Annexin V and propidium iodide staining.

1 × 10^5^ to 5 × 10^5^ cells/condition were collected, centrifuged (400*g* for 5 minutes), and washed once with PBS. Pellets were resuspended in 500 μL Annexin V APC staining solution (Abcam, ab236215) and incubated in the dark at room temperature for 10 minutes. Samples were centrifuged (400*g* for 5 minutes), resuspended in 200 μL 50 μg/mL Propidium Iodide solution (Invitrogen, P3566) in PBS, and incubated in the dark at room temperature for 10–15 minutes. Following incubation, 200 μL PBS was added into each tube. Samples were analyzed using CytoFLEX (Beckman Coulter Inc.) and FlowJo Software.

### Multiplex and HALO analysis.

Multiplex immunofluorescence staining of AKPT tumors was performed on 4 μm thick FFPE sections using the OPAL protocol (Akoya Biosciences). The Leica BOND RXm autostainer (Leica Microsystems) was used to conduct this staining. Staining cycles were conducted 6 consecutive times using the following primary antibody-Opal fluorophore pairs for the immune panel: (a) Ly6G (1:300, 551459; BD Pharmingen) Opal 540; (b) CD4 (1:500, ab183685; Abcam) Opal 520; (c) CD8 (1:800, 98941; Cell Signaling) Opal 570; (d) CD68 (1:1200, ab125212; Abcam) Opal 620; (e) FoxP3 (1:400, 126553; Cell Signaling) Opal 650; and (f) E-cadherin (1:500, 3195; Cell Signaling) Opal 690. The following primary antibody-Opal fluorophore pairs were used for the stroma panel: (a) Gremlin 1 (1:750, AF956; R&D Systems) Opal 540; (b) CD34 (1:3,000, ab81289; Abcam) Opal 520; (c) CD146 (1:500, ab75769; Abcam) Opal 570; (d) SMA (1:1000, ab5694; Abcam) Opal 620; (e) Periostin (1:1000, ab227049; Abcam) Opal 690; and (f) E-cadherin (1:500, 3195; Cell Signaling) Opal 650.

Tissues sections were incubated for 1 hour in primary antibodies and detected using the BOND Polymer Refine Detection System (DS9800; Leica Biosystems) in accordance with the manufacturer’s instructions, substituting DAB for the Opal fluorophores, with a 10-minute incubation time and withholding the hematoxylin step. Antigen retrieval at 100°C for 20 minutes, in accordance with standard Leica protocol, with Epitope Retrieval Solution one or two (AR9961; Leica Biosystems) was performed prior to each primary antibody being applied. Sections were then incubated for 10 minutes with spectral DAPI (FP1490, Akoya Biosciences), and the slides were mounted with VECTASHIELD Vibrance Antifade Mounting Medium (H-1700-10; Vector Laboratories). Whole slide scans and multispectral images (MSI) were obtained on the Akoya Biosciences Vectra Polaris. Batch analysis of the MSIs from each case was performed with the inForm 2.4.8 software provided. Finally, batched analyzed MSIs were fused in HALO (Indica Labs) to produce a spectrally unmixed reconstructed whole-tissue image. Cell density analysis was performed for each cell phenotype across the 3 MPIF panels using HALO. HALO Image Analysis Platform version 3.5.3577 and HALO AI version 3.5.3577 (Indica Labs Inc.) were used. Analysis modules “Area Quantification 2.4.2”, “HighPlex FL 4.2.3” and “Random Forest Classifier” were used.

Cover slips were lifted after multiplex staining and C5aR1 (1:1000, Abcam, catalog ab59390) was stained for chromogenically on the Leica BOND autostainer. Antigen retrieval was carried out at 100°C for 20 minutes with Epitope Retrieval Solution two. Primary antibody incubation at 1:250 dilution for 30 minutes was followed by detection using the BOND Polymer Refine Detection System (DS9800, Leica Biosystems) as per manufacturer’s instructions.

### Immunoblotting.

Cells were lysed in UTB (9 M urea; 75 mM Tris-HCl, pH 7.5; and 0.15 M β-mercaptoethanol) and sonicated briefly before quantification, as described in detail (https://data.mendeley.com).

For intestinal organoid experiments, harvesting was carried out in Corning Cell Recovery Solution at 4°C for 30 minutes. Samples were then washed with cold PBS, pelleted (700*g*, 3 minutes, 4°C), and lysed immediately in RIPA buffer (Sigma-Aldrich) containing 1:100 protease inhibitor cocktail (Sigma-Aldrich) and 1:100 phosphatase inhibitor cocktail two (Sigma-Aldrich). Proteins were quantified with the Pierce BCA Protein Assay Kit (Thermo Fisher Scientific), using a BSA standard curve (20–2,000 μg/mL).

4%–20% polyacrylamide gels (Bio-Rad) were used for protein separation. Proteins were blotted onto a nitrocellulose membrane (Bio-Rad). The Bio-Rad Chemidoc XRS system or LI-COR Odyssey imaging system was used. In each case, experiments were carried out in triplicate, and a representative blot is shown unless otherwise stated. See supplemental material for full, uncut gels.

Antibodies used were was follows: β-actin (Sigma-Aldrich, A5441), hFAB Rhodamine Anti-Tubulin (Bio-Rad, 12004166), RelA/p65 (Cell Signaling, 3034), pRelA/p65-S536 (Cell Signaling, 3033T), total AKT (Cell Signaling, 2920), AKT-T308 (Cell Signaling, 13038), p44/p42 (Cell Signaling, 4696), phospho p44/p42 (Cell Signaling, 4376), and γH2AX (Millipore, 05-636-1).

### RPPA.

RPPA was performed by the University of Texas MD Anderson RPPA core as described in a published protocol (https://www.mdanderson.org/research/research-resources/core-facilities/functional-proteomics-rppa-core.html). For statistical analysis on differently expressed proteins, linearized (standard curve) normalized (to protein loading) relative protein levels were analyzed using 2-tailed Student’s *t* test (t.test()) in R.

### Immunofluorescence.

Staining was carried out as previously described (41). For 53BPI foci studies, 53BP1 antibody (Cell Signaling Technology, 4937) and secondary anti-Rabbit, Alexa Fluor 594 (Thermo Fisher Scientific, A32754) were used. For C5aR1-GFP overexpression studies, cells were transfected with C5AR1_OHu107216C_pcDNA3.1(+)-C-eGFP or empty vector (GenScript) using Lipofectamine 3000 as per the manufacturer’s instructions (Invitrogen). 24 hours later slides were fixed in 4% PFA for 15 minutes at room temperature. Phalloidin 647 (1:1,000 dilution, A22287) was added to each coverslip and left in a 37°C oven for 1 hour in a humidified chamber. For both 53BP1 foci and C5aR1 overexpression studies, coverslips were mounted onto microscope slides using ProLong Mounting Medium with DAPI Stain (P36931, Thermo Fisher Scientific) and were imaged on the Zeiss LSM 710 Confocal Microscope.

### Flow cytometry analyses.

One week after RT, the mice were sacrificed and tumor draining lymph nodes were collected. Cells were incubated with anti-FcR antibody (clone 24G2) and stained with the following surface antibodies: anti-CD45.2 (clone 104, eBioscience), CD3 (clone 17A2, eBioscience), CD4 (cloneRM4-5), CD8a (clone 53-6-7), B220 (clone RA3-6B2), CD25 (clone PC61), a-CD49b (clone DX5), anti–IFN-γ (clone XMG1.2), anti-FoxP3 (clone FJK-16s, eBioscience), anti–TNF-α (clone MP6-XT22), anti-Granzyme B (clone BG11, BioLegend). Dead cells were stained using a Fixable Viability Dye eFluor 506 (eBioscience). For cytokine staining, cells were stimulated for 4 hours with 50 ng/mL PMA and 500 ng/mL Ionomycin (Sigma-Aldrich), washed, and after surface staining, permeabilized with Cytofix/Cytoperm buffer according to manufacturer instructions. FoxP3 permeabilization buffer (eBioscience) was used for FoxP3 staining. All antibodies were purchased from BD Pharmingen unless otherwise specified. Samples were acquired with Fortessa LSR (BD Bioscience) and analyzed with FlowJo software.

For analysis of tumor immune infiltration, female C57BL/6 MC38 tumor tissue was digested into a single suspension using the murine tumor dissociation kit from Miltenyi Biotech as per the manufacturer’s protocol. After RBC lysis, cells were resuspended in PBS, counted, and then stained with Zombie NIR (BioLegend) for live/dead cell discrimination. Nonspecific binding was blocked using an anti-mouse CD16/32 (BioLegend) antibody, following which, cell surface staining was performed using fluorophore-conjugated anti-mouse CD45.1 (30-F11), CD11b (M1/70), CD11c (N418), Ly6G (1A8), Ly6C (HK1.4), and CD8 (53-6.7), all from Biolegend. Cell acquisition was performed with FACSDiva software on an LSR II flow cytometer (BD Biosciences) and analyzed with FlowJo software. Compensations were attained using anti-rat and anti-hamster compensation beads (BD Biosciences). For fixable live/dead staining, compensation was performed using ArC amine reactive compensation beads (BD Biosciences).

The gating schemes of dissociated tissues were as follows: immune cells (ZNIR^−^CD45^+^), CD8 T cells (ZNIR^−^CD45^+^CD8^+^), neutrophils (ZNIR^−^CD45^+^CD11b^+^Ly6G^+^), and monocytes (ZNIR^−^CD45^+^CD11b^+^Ly6C^+^).

For C5aR1 expression analysis in cell lines, 1 × 10^6^ cells (either HCT116 or MC38 as indicated in figures) were collected into each tube, centrifuged at 400*g* for 5 minutes, and washed once with PBS. Pellets were resuspended in 100 μL Zombie Green Fixable Viability Kit (BioLegend, 423111) or Zombie NIR (BioLegend, 423105) diluted 1:5,000 and incubated in the dark at room temperature for 15–30 minutes. Following incubation, cells were washed once in buffer containing 0.5% BSA. Cells were resuspended in PE/Cyanine7 anti-mouse CD88 antibody (BioLegend, 135810) or BD OptiBuild BV421 Mouse Anti-Human CD88 (BD Biosciences, 742315) at 1 μg per million cells in 100 μL volume. PE/Cyanine7 Rat IgG2b, κ Isotype Ctrl Antibody (BioLegend, 400618) and BD Horizon BV421 Mouse IgG1, k Isotype Control (BD Biosciences, 562438) were also used at 1 μg per million cells in 100 μL volume. Samples were analyzed using CytoFLEX (Beckman Coulter Inc.). Data analysis was done using FlowJo Software (version 10.7.2).

### qRT-PCR.

RNA was extracted using Trizol (Invitrogen/Life Technologies, 15596018). The iScript cDNA synthesis kit (Bio-Rad, 1708891) or Verso cDNA Synthesis kit (Thermo Fisher Scientific, AB-1453/B) was used to reverse transcribe cDNA from total RNA according to manufacturer’s instructions. Relative mRNA levels were calculated using the dCt methodology using a 7900HT Fast Real-Time PCR System. Primers used were as follows: ACTB forward, ACATCCGCAAAGACCTCTACG, ACTB reverse, TTGCTGATCCACATCTGCTGG; C5AR1 forward, TCCTTCAATTATACCACCCCTGA, C5AR1 reverse, ACGCAGCGTGTTAGAAGTTTTAT; BCL2 forward, TTGCCAGCCGGAACCTATG, BCL2 reverse, CGAAGGCGACCAGCAATGATA; C5 forward, CTCCTCAGGCCATGTTCATT; C5 reverse, TCTTTTGGCTGGCTTCAAGT; 18s forward, GTGGAGCGATTTGTCTGGTT, 18s reverse, ACGCTGAGCCAGTCAGTGTA; C5ar1 forward, ACATGGACCCCATAGATAAC, C5ar1 reverse, ACCACCGAGTAGATGATAAG; c5 forward, TACCAATGCCAACCTGGTGAAAGG, c5 reverse, TCTGCAGAACCTCTTTGCCCATGA; Bcl2 forward, GTCGCTACCGTCGTGACTTC, Bcl2 reverse, CAGACATGCACCTACCCAGC, Bcl2l1 forward, GACAAGGAGATGCAGGTATTGG, bcl2l1 reverse, TCCCGTAGAGATCCACAAAAGT, and Xiap1 forward, CGAGCTGGGTTTCTTTATACCG, Xiap1 reverse, GCAATTTGGGGATATTCTCCTGT.

### siRNA transfection.

NFKBIA (L-004765-00), RelA (L-003533-00), C5aR1 (L-005442-00), or nontargeting RNAi negative control (Scramble, D-001810-10) (all from Dharmacon) were transfected into HCT116 cells using Lipofectamine RNAiMax transfection reagent (Invitrogen, 13778075) at a final concentration of 50 nM, according to the manufacturers’ instructions. Cells were harvested 72 hours after transfection.

### In silico screen.

Data were accessed through the DepMap portal (https://depmap.org/portal/, September 10, 2020). Essentiality scores were calculated by dividing the number of dependent cell lines for each gene/total number of cell lines in either CRISPR/Cas9 and RNAi screens. To identify stress- and cancer-specific dependencies, we looked for genes that were deemed nonessential across all cell lines (genes that when knocked out/down under baseline conditions do not alter cell survival) while still being expressed across cell lines. Genes described as complement system components, receptors, proteases, and regulators (as reported in ref. 17) were queried. The calculated essentiality score was presented as a color in the heatmap. *ATR* was included in the screen a positive control. Target tractability was assessed by accessing CanSAR data, as displayed on the DepMap portal. A gene was only considered a hit if it was “druggable” based on structural and ligand-based assessment. C1R and CF1 were not included in the heatmap due to lack of CRISPR/Cas9 or RNAi data. C4A, C4B, VSIG4, C8A, C8B, CD93, and CR1 were not included due to their very low expression in the majority of tissues.

### RNA-Seq.

Transcriptomic profiling of mouse tumor tissues was carried out by 3′RNA-Seq. Extracted RNA was quantified using RiboGreen (Invitrogen) on the FLUOstar OPTIMA plate reader (BMG Labtech), and the size profiles and integrity were analyzed on the TapeStation (Agilent, RNA ScreenTape). Libraries were prepared using the Lexogen QuantSeq 3′mRNA-Seq kit FWD kit (Lexogen, 015.2 × 96) and the Lexogen UMI Second strand synthesis module for QuantSeq FWD (Lexogen, 81.96) following the manufacturer’s instructions. Individual libraries were quantified using a Qubit Fluorometer (Invitrogen), and the size profiles were analyzed on the Agilent TapeStation. Individual libraries were normalized and pooled accordingly to multiplex for sequencing. Libraries were sequenced on a NextSeq 500 instrument (Illumina) as 75 bp single-end reads.

Raw sequence reads were subjected to adapter trimming using *BBduk* (BBTools ver. 38.46) Trimmed reads were aligned to the Genome Reference Consortium mouse genome build 38 (GRCm38) of the mouse reference using *STAR* (ver. 2.7.0f). Ensembl 96 annotations were used for alignment and subsequent quantifications. Gene expression was quantified using *featureCounts* (ver. 1.6.4). Further analyses of RNA-Seq data were carried out in the R statistical environment (ver. 4.0.3). Differential expression analyses were performed using the *limma* (ver. 3.46) package. Gene set enrichment analyses (GSEA) were performed using the *fgsea* package (ver. 1.16).

### Organoids RNA extraction for sequencing.

Organoids were harvested by direct incubation in 350 μL RLT plus lysis buffer (Qiagen RNeasy Plus Micro kit) for 5 minutes. RNA was extracted using the Qiagen RNeasy Plus Micro kit according to the manufacturer’s protocol. RNA quality and concentration were measured with Bioanalyzer 2100 (Agilent) and Nanodrop (Thermo Fisher Scientific), respectively. RNA library preparation, sequencing, and data analysis were outsourced to Novogene.

### CMS in rectal tumors.

The Grampian cohort profiled by the S:CORT consortium was used. Pretreatment rectal tumor biopsies from patients treated with radiotherapy and capecitabine were selected (*n* = 129). Xcell array data were normalized and CMS was called using the R tool CMScaller (42). Patients gave consent to biobank their samples in the Grampian Biorepository (ref no. TR000028, 1/10/2014) with release of linked anonymized clinical data for ethically approved research projects.

### Analysis on longitudinal biopsies.

Patients received nCRT as part of their standard-of-care management. The gross rectal tumor and tumor involved lymph nodes were irradiated to a dose 50 Gy in 25 fractions, and pelvis nodes to a dose of 45 Gy in 25 fractions, over 5 weeks, concurrently with capecitabine chemotherapy at 900 mg/m^2^. Patients underwent sigmoidoscopy at the described time points with direct visualization and biopsies of the tumor.

### RNA-Seq of longitudinal biopsies.

The Partek Flow server was used to process FASTQ files by adapter trimming, quality control alignment to the genome, and abundance estimation. R version 4.1.2. was used to perform all exploratory analysis. R package “DESeq2” was used to calculate differentially expressed genes between baseline and after treatment time points (43). Genes with *P* values of less than 0.05 were considered significantly different between time points, and a positive log_2_ fold change was indicative of a gene with greater expression after the start of treatment. Differentially expressed genes were visualized using a custom volcano plot produced using “ggplot2”; complement-associated genes were annotated on the volcano plot (17). Genes ranked by Wald statistic were used to perform pairwise GSEA using the “fgsea” package, mSigDB Hallmark signature set, and the complement signature from Ricklin et al. 2010 (17, 44, 45). To gauge the relative enrichment of the “Hallmark Complement” gene set, the signatures within the mSigDB Hallmark set were ranked by normalized enrichment score.

### Staining and analysis of longitudinal biopsies.

C5aR1 staining was performed as above. For analysis, the Indica Labs HALO image analysis software (v3.6.4134.137 and HALO AI 3.6.4134) was used. Sections were manually annotated by a trained pathologist as cancer, normal/reactive, or ulcer/granulation tissue. DenseNet V2 (HALO AI) was then “taught” to discriminate among epithelium, stroma, and glass. Within each annotation region, stroma and epithelium tissue compartments were analyzed separately for (a) general tissue DAB intensity using “Indica Labs Area Quantification v2.4.2” and (b) cell localized DAB intensity using “Indica Labs Multiplex IHC v3.4.9.” Both analyses yielded the same overall results. H-scores are presented in this manuscript.

### Statistics.

Statistical analysis was carried out using Graphpad Prism software (version 9.2.0). *P* values were used to determine significance of differences, and *P* values of less than 0.05 were considered significant. Two-tailed Student’s *t* test and 1- or 2-way ANOVA were used as appropriate and as specified in each figure legend.

### Study approval.

This study involved human participants, and all samples were obtained following individual informed consent for research projects and subsequent ethical approval by the National Research Ethics Service in the UK (REC ref. 15/EE/0241, IRAS ref 169363, and REC ref 18WS00031). Experimental procedures were carried out under a project license issued by the UK Home Office under the UK Animals (Scientific Procedures) Act of 1986; under approved protocols by the Institutional Animal Care and Use Committee at Medical University of South Carolina; or in accordance with the NIH guidelines for the use and care of live animals (approved by the Stanford University Institutional Animal Care and Use Committee). Animal Research Reporting of In vivo Experiments guidelines were used.

### Data availability.

The RNA-Seq data generated for this paper have been deposited at the ArrayExpress database at EMBL-EBI (https://www.ebi.ac.uk/biostudies/arrayexpress) and are publicly available. The data from the time-course study after radiation treatment of heterotopic AKPT organoid-derived tumors are available under accession E-MTAB-12538, and the data from the intestinal organoids treated with PMX205 and/or RT are available under accession E-MTAB-12548. Publicly available data were analyzed in this paper. The analysis of WT, AKPT, and KPN intestinal organoids used data deposited at the ArrayExpress database under accession E-MTAB-11769 (46). Underlying data can also be accessed in the Supporting Data Values file or from the corresponding author upon request.

## Author contributions

CB, D MacLean, D Majorova, SM, DKN, RKK, SG, GNV, MDD, TS, AE, DJ, YJ, ED, and MMO provided methodology. MMO wrote the original draft of the manuscript. MMO, D MacLean, AJG, DKN, SG, GNV, MDD, AE, ED, EJM, YJ, SJAB, MS, SJL, QTL, TMW, and SMO reviewed and edited the manuscript. MMO and AJG conceptualized the study. CB, D MacLean, D Majorova, SM, DKN, RKK, SG, CK, GNV, TS, MDD, ED, YJ, EJM, DJ, AKM, KP, and MMO provided investigation. MMO, DKN, RKK, CB, D MacLean, D Majorova, ED, DJ, SG, GNV, LVSH, and RKM provided formal analysis. MMO, AJG, TMW, SJAB, SJL, QTL, EGG, SG, ACK, CSR, and MS provided resources. MMO, AJG, SJL, TM, QTL, SMO, JE, and CSR provided supervision. MMO, AJG, SJL, CSR, and MS acquired funding. Co–first authors are listed in alphabetical order.

## Supplementary Material

Supplemental data

Supplemental tables 1-6

Supporting data values

## Version 1 10/12/2023

In-Press Preview

## Version 2 12/01/2023

Electronic publication
