# Improving radiotherapy in immunosuppressive microenvironments by targeting complement receptor C5aR1

**DOI:** 10.1172/JCI185067

**Published:** 2024-08-15

**Authors:** Callum Beach, David MacLean, Dominika Majorova, Stavros Melemenidis, Dhanya K. Nambiar, Ryan K. Kim, Gabriel N. Valbuena, Silvia Guglietta, Carsten Krieg, Mahnaz Darvish-Damavandi, Tatsuya Suwa, Alistair Easton, Lily V.S. Hillson, Ashley K. McCulloch, Ross K. McMahon, Kathryn Pennel, Joanne Edwards, Sean M. O’Cathail, Campbell S. Roxburgh, Enric Domingo, Eui Jung Moon, Dadi Jiang, Yanyan Jiang, Qingyang Zhang, Albert C. Koong, Trent M. Woodruff, Edward E. Graves, Tim Maughan, Simon J.A. Buczacki, Manuel Stucki, Quynh-Thu Le, Simon J. Leedham, Amato J. Giaccia, Monica M. Olcina

Original citation: *J Clin Invest*. 2023;133(23):e168277. https://doi.org/10.1172/JCI168277

Citation for this corrigendum: *J Clin Invest*. 2024;134(16):e185067. https://doi.org/10.1172/JCI185067

During the preparation of this manuscript, the dose for PMX205 used for in vitro experiments was stated incorrectly in the legend for Figure 3A and in the Methods. In addition, the statement in Methods regarding staining for C5aR1 using the Leica BOND autostainer was incorrect. The correct sentences are below. The HTML and PDF files have been updated.

(**A**) HCT116 cells were treated with either vehicle or PMX205 (10 μg/mL) for 48 hours.

*PMX205 treatment*. For all in vitro experiments, cells were treated with 10 μg/mL PMX205 (Tocris, 5196) dissolved in 20% ethanol/water.

*Proliferation assays*. HCT116 or MC38 cells were seeded at the indicated concentration. Cells were treated with 10 μg/mL PMX205 (or RNAse-Free water as vehicle). On day 2, cells were counted using a hemocytometer and trypan blue in a 1:1 ratio. On day 3, 10 μg/mL PMX205 (or water) was added to the dishes.

C5aR1 (Abcam, catalog ab59390) was stained for chromogenically on the Leica BOND autostainer on a serial section.

The authors regret the errors.

## Version 1 08/15/2024

Electronic publication

