# Supplementary material for: Improving radiotherapy in immunosuppressive microenvironments by targeting complement receptor C5aR1
Source: J Clin Invest. 2023 Dec 1;133(23):e168277. doi: 10.1172/JCI168277 (PMC10688992; doi:10.1172/JCI168277)

Supplementary Information

Improving radiotherapy in immunosuppressive microenvironments by targeting complement receptor C5aR1

Beach Callum[1+], MacLean David[1+], Majorova Dominika[1+], Melemenidis Stavros[2], Nambiar Dhanya K.[2], Kim Ryan K.[2], Valbuena Gabriel N.[3], Guglietta Silvia[4,5], Krieg Carsten[5,6], Darvish Damavandi Mahnaz[7], Suwa Tatsuya[1], Easton Alistair[1], Hillson Lily V.S.[8], McCulloch Ashley K.[8], McMahon Ross K.[8], Pennel Kathryn[8], Edwards Joanne[8], O'Cathail Sean M.[8], Roxburgh Campbell S.[8], Domingo Enric[1], Moon Eui Jung[1,2], Jiang Dadi[9], Jiang Yanyan[1], Zhang Qingyang[1], Koong Albert C[9], Woodruff Trent M.[10], Graves Edward E.[2], Maughan Tim[1], Buczacki Simon J. A.[7], Stucki Manuel[11], Le Quynh-Thu[2], Leedham Simon J.[3], Giaccia Amato J.[1,2] and Olcina Monica M.[1,2,11*]

1. Department of Oncology, University of Oxford, Old Road Campus Research Building, Roosevelt Drive, Oxford, OX3 7DQ. UK.
2. Department of Radiation Oncology, Stanford University, Stanford, CA 94305, USA.
3. Wellcome Centre for Human Genetics, Roosevelt Drive, University of Oxford, Oxford UK.
4. Department of Regenerative Medicine and Cell Biology, Medical University of South Carolina, 29425 Charleston, SC, USA
5. Hollings Cancer Center, Medical University of South Carolina, 29425 Charleston, USA
6. Department of Pathology and Laboratory Medicine, Medical University of South Carolina, 29425 Charleston, SC, USA
7. Nuffield Department of Surgical Sciences, University of Oxford, Headington, Oxford, OX3 7DQ, UK.
8. School of Cancer Sciences, University of Glasgow, UK
9. The UT MD Anderson Cancer Center, 6565 MD Anderson Blvd, Z6.3030, Zayed Building, Houston, Texas, 77030, USA
10. School of Biomedical Sciences, Faculty of Medicine, The University of Queensland, Brisbane, 4072, Australia.
11. Department of Gynecology, University of Zurich, Wagistrasse 14, 8952 Schlieren, Switzerland.

+ Joint author.

*Corresponding Author:

Monica M. Olcina
Current address:
Oxford Institute of Radiation Oncology
University of Oxford, Old Road Campus Research Building
Roosevelt Drive, Oxford, OX37DQ.

Running tittle: Targeting C5aR1 to improve radiotherapy in immune excluded tumours

Keywords: C5aR1, colorectal cancer, radiotherapy, complement

Supplementary Figure 1. Identification of radiation-responsive targets in immunosuppressive tumours

- **(A)** Machine learning-based quantification of CD4+ cells present in AKPT tumours following multiplex staining at different timepoints following RT. n=5/group. * = $p < 0.05$, ns = not significant (p=>0.05) by ordinary one-way ANOVA with Tukey's multiple comparisons.
- **(B)** Machine learning-based quantification of Treg cells present in AKPT tumours following multiplex staining at different timepoints following RT. n=5/group. ** = $p < 0.01$, ns = not significant (p=>0.05) by ordinary one-way ANOVA with Tukey's multiple comparisons.
- **(C)** Machine learning-based quantification of CD8+ cells present in AKPT tumours following multiplex staining at different timepoints following RT. n=5/group. * = $p < 0.05$, ns = not significant (p=>0.05) by ordinary one-way ANOVA with Tukey's multiple comparisons.
- **(D)** Machine learning-based quantification of neutrophils present in AKPT tumours following multiplex staining at different timepoints following RT. n=5/group. **** = $p < 0.0001$, * = $p < 0.05$, ns = not significant (p=>0.05) by ordinary one-way ANOVA with Tukey's multiple comparisons.
- **(E)** Machine learning-based quantification of macrophages present in AKPT tumours following multiplex staining at different timepoints following RT. n=5/group. Machine learning-based quantification of immune cell infiltration in epithelial regions of AKPT tumours following multiplex staining at different timepoints following RT. ** = $p < 0.01$, ns = not significant (p=>0.05) by ordinary one-way ANOVA with Tukey's multiple comparisons.
- **(F)** Machine learning-based quantification of immune cell infiltration in epithelial regions of AKPT tumours following multiplex staining at different timepoints following RT.
- **(G)** Machine learning-based quantification of immune cell infiltration in stromal regions of AKPT tumours following multiplex staining at different timepoints following RT.
- **(H)** Pairwise geneset enrichment analysis comparing irradiated AKPT tumours at 4 hours, 24 hours, 3 days or 7 days, post 15 Gy compared to unirradiated controls. P-values, Enrichment Scores (ES) and NES scores are also provided for Hallmark complement signatures.
- **(I)** Graph shows C3 expression at different timepoints following RNA-seq of AKPT tumour receiving 15 Gy.
- **(J)** Graph shows Cfp expression at different timepoints following RNA-seq of AKPT tumour receiving 15 Gy.
- **(K)** Graph shows Cfi expression at different timepoints following RNA-seq of AKPT tumour receiving 15 Gy.

- **(L)** Graph shows C1qbp expression at different timepoints following RNA-seq of AKPT tumour receiving 15 Gy.
- **(M)** Graph shows Serping1 expression at different timepoints following RNA-seq of AKPT tumour receiving 15 Gy.
- **(N)** Pairwise gene set enrichment analysis comparing baseline samples to samples collected 2-weeks, 6-weeks or 12-weeks after starting RT in longitudinal biopsies from rectal adenocarcinoma patients. P-values, Enrichment Scores (ES) and NES scores are also provided for Hallmark complement signatures.

Supplementary Figure 2: C5aR1 is a radiation-responsive druggable target

(A) Prognoscan KM curve for CRC patients with high (red) or low (blue) C5AR1 mRNA expression levels is shown. This analysis was based on the PrognoScan database (http://www.prognoscan.org/) using the publicly available Gene Expression Omnibus (http://www.ncbi.nlm.nih.gov/geo).
(B) C5aR1 expression following RNA-seq of AKPT tumour receiving either 15 Gy or 3 x 7 Gy . * = $p<0.05$.
(C) Immunohistochemistry staining of a small intestine section of a C5aR1$^{-/-}$ mouse used as a negative control for C5aR1 staining.
(D) C5AR1 mRNA expression in pre-treatment rectal tumour biopsies (N=129) classified according to consensus molecular subtypes (CMS). Significance assessed by Wilcoxon test.
(E) Volcano plot of differentially expressed genes (baseline vs week 12) in longitudinal biopsies from rectal adenocarcinoma patients undergoing long-course chemoradiotherapy.

(F) C5aR1 expression following RNA-seq in longitudinal biopsies from rectal adenocarcinoma patients taken at baseline, 2-weeks, 6-weeks or 12-weeks following long-course chemoradiotherapy. Corresponding p-values are shown at the top of the graph.
(G) Workflow of analysis of data presented in Figure 2I and Supplementary Figure 2H.
(H) H-Scores of C5aR1 staining in epithelial and stromal areas of normal/reactive tissue from rectal adenocarcinoma longitudinal biopsies taken at baseline (W0) compared to week 2 (W2) following long-course chemoradiotherapy.
(I) mRNA expression of *c5ar1/housekeeping* in MC38 cells treated with either 0 or 9 Gy . Individual points indicate average from biologically independent replicates. n=3. * = $p<0.05$, two-tailed t-test.
(J) mRNA expression of *c5/housekeeping* in MC38 cells treated with either 0 or 9 Gy. Individual points indicate average from biologically independent replicates. n=3. ns = not significant (p=>0.05).
(K) mRNA expression of *C5AR1/housekeeping* in HT29 cells treated with either 0 or 9 Gy. n=3.Individual points indicate technical replicates. ** = $p<0.01$, two-tailed t-test.
(L) mRNA expression of *C5/housekeeping* in HT29 cells treated with either 0 or 9 Gy. n=3. Individual points indicate technical replicates. ** = $p<0.01$, two-tailed t-test.
(M) C5aR1 Mean Fluorescence Intensity in MC38 cells treated with either 0 or 9 Gy. Individual points indicate average from biologically independent replicates. n=3, ** = $p<0.01$, two-tailed t-test.

(N) Representative confocal microscopy images of cells transfected with either empty vector or GFP-tagged C5aR1. Phalloidin is show in magenta and DAPI in blue.

Supplementary Figure 3: C5aR1 regulates tumour cell survival under stress

- **(A)** MC38 cells were treated with either vehicle or PMX205 (10 μg/ml) for 48 hours. Western blotting was carried out with the antibodies indicated. n=3.
- **(B)** HT29 cells were treated with 0 or 9 Gy and either vehicle or PMX205 (10 μg/ml) 1 hour before RT. Cells were harvested 48 hours post-RT. Western blotting was carried with the antibodies indicated. β-actin was used as the loading control. n=3.
- **(C)** HCT116 cells were treated with 0 or 9 Gy and either vehicle or PMX205 (10 μg/ml) 1 hour before RT. Cells were harvested 48 hours post-RT. Western blotting was carried with the antibodies indicated. β-actin was used as the loading control. n=3.
- **(D)** HCT116 cells were treated with 0 or 9 Gy and either vehicle or PMX205 (10 μg/ml) 1 hour before RT. Cells were harvested 6 or 24 hours post-RT. Percentage of cells in each cell cycle phase is shown. n=2. Error bars indicate range.
- **(E)** HT29 cells were treated with 0 or 9 Gy and either vehicle or PMX205 (10 μg/ml) 1 hour before RT. Cells were harvested 6 or 24 hours post-RT (or following 0 Gy treatment). Percentage of cells in each cell cycle phase is shown. n=2. Error bars indicate standard deviation.
- **(F)** HCT116 cells were treated with either vehicle or PMX205 (10 μg/ml). Cell number following treatment was assessed at the indicated timepoints. n=3. Differences between vehicle and PMX205 are not-significant (by multiple paired t-test) at all timepoints. Error bars indicate standard deviation.
- **(G)** MC38 cells were treated with either vehicle or PMX205 (10 μg/ml). Cell number following treatment was assessed at the indicated timepoints. n=3. Differences between vehicle and PMX205 are not-significant (by multiple paired t-test) at all timepoints. Error bars indicate standard deviation.
- **(H)** HCT116 cells were treated with 0 or 9 Gy and either vehicle or PMX205 (10 μg/ml) 1 hour before RT. Cells were harvested either 6 or 48 hours post-RT. Western blotting was carried with the antibodies indicated. β-actin was used as the loading control.
- **(I)** HCT116 cells were treated with 0 or 4 Gy and either vehicle or PMX205 (10 μg/ml) 1 hour before RT. Cells were fixed at the indicated timepoints post-RT. Graph shows the normalised foci per cell. n=3. ns = not significant (p=>0.05) by two-way ANOVA, multiple comparisons.
- **(J)** Heatmap of proteins differentially expressed following reverse phase protein array (RPPA) analysis in HCT116 cells treated with vehicle or PMX205. n=3.
- **(K)** The graph represents the number of dead (apoptotic) cells expressed as a % of the whole population for HCT116 cells treated with either vehicle or PMX205 (10 μg/ml) for 1 hour before treatment with either 0 or 9 Gy. Cells were harvested 48 hours post-RT. n=3.

(L) The graph represents the number of either live or dead (necrotic, early or late apoptotic) cells expressed as a % of the whole population for MC38 cells treated with either vehicle or PMX205 (10 μg/ml) and either 0 or 9 Gy. Cells were harvested 48 hours post-RT. n=2.

(M) The graph represents the number of dead (apoptotic) cells expressed as a % of the whole population for HCT116 cells treated with either Scr or C5aR1 siRNA and either 0 or 9 Gy RT. Cells were harvested 48 hours post-RT. Counted fields of view from all experiments are shown. n=3.

(N) mRNA expression of *C5aR1/β-actin* in HCT116 cells treated with either scramble or C5aR1 siRNA. Data from one representative experiment is shown.

(O) The graph represents the number of dead (apoptotic) cells expressed as a % of the whole population for HCT116 cells treated with either vehicle, PMX205, 5-FU or a combination of PMX205 and 5-FU. Counted fields of view from all experiments are shown. n=3.

(P) The graph represents the number of dead (apoptotic) cells expressed as a % of the whole population for HCT116 cells treated with either vehicle, PMX205, Oxaliplatin or a combination of PMX205 and Oxaliplatin. Counted fields of view from all experiments are shown. n=3.

(Q) The graph represents the number of dead (apoptotic) cells expressed as a % of the whole population for HCT116 cells treated with either scr or IκBα siRNA and either 0 or 9 Gy. Cells were harvested 48 hours post-RT. Counted fields of view from all experiments are shown.

(R) HCT116 cells treated with 9 Gy and either scr, IκBα siRNA and vehicle or PMX205. Western blotting was carried with the antibodies indicated. β-actin was used as the loading control.

(S) The graph represents the number of dead (apoptotic) cells expressed as a % of the whole population for HCT116 cells transfected with either scr or RelA siRNA and treated with either vehicle or PMX205 for 1 hour before RT with either 0 or 9 Gy. Cells were harvested 48 hours post-RT. Independent fields of view from a representative experiment are show, n=2.

(T) HCT116 cells treated with either scr or RelA siRNA. Western blotting was carried with the antibodies indicated. β-actin was used as the loading control.

(U) mRNA expression of *BCL2/housekeeping* in HCT116 cells treated with either vehicle or PMX205 and 0 or 9 Gy. n=3. *** = p<0.001, two-tailed t-test.

(V) The graph represents the ratio of dead (apoptotic) cells expressed as a % of the whole population relative to those cells treated with vehicle (9 Gy) for HCT116 cells treated with either PMX205, Selumetinib or a combination of PMX205 and selumetinib and 9 Gy. Cells were harvested 48 hours post-RT. n=3.

(W) The graph represents the number of dead (apoptotic) cells expressed as a % of the whole population for HCT116 cells treated with either PMX205, Selumetinib or a combination of PMX205 and selumetinib and either 0 or 9

Gy. Cells were harvested 48 hours post-RT. n=3. Counted fields of view from all experiments are shown. n=3.

Supplementary Figure 4: C5aR1 deficiency does not result in increased apoptosis in healthy intestinal epithelium

- **(A)** Murine intestinal organoids were treated with 0 or 9 Gy and either vehicle or PMX205 1 hour before RT. Western blotting was carried with the antibodies indicated. Total Erk1/2 was used as the loading control.
- **(B)** GeneRatios are shown following GO enrichment analysis of murine intestinal organoids treated +/- PMX205 +/- 9 Gy. Organoids were harvested 48 hours post-RT. Oxidoreductase activity, acting on … = oxidoreductase activity, acting on single donors with incorporation of molecular oxygen; oxidoreductase activity, acting on single donors with incorporation of molecular oxygen, incorporation of two atoms of oxygen.
- **(C)** GeneRatios are shown following KEGG (Kyoto Encyclopedia of Genes and Genomes) pathway analysis in murine intestinal organoids treated +/- PMX205 +/- 9 Gy. Organoids were harvested 48 hours post-RT. Glycosphingolipid biosynthesis – lacto… = Glycosphingolipid biosynthesis - lacto and neolacto series. Glycosaminoglycan biosynthesis – chondroitin… = Glycosaminoglycan biosynthesis - chondroitin sulfate / dermatan sulfate. Glycosphingolipid biosynthesis – globo… = Glycosphingolipid biosynthesis - globo and isoglobo series.
- **(D)** Table summarising gene expression changes following RNA-seq of mouse intestinal untransformed WT organoids treated with either 9 Gy vs PMX205 + 9 Gy RT (organoids harvested 48 hours following RT). n=3 biological replicates.

Supplementary Figure 5: C5aR1 inhibition improves tumour radiation response

- **(A)** Graph shows the weight of mice treated with 9 Gy single dose RT and either vehicle or PMX205 for 3 doses flanking RT (on day 0, 1 and 2). Individual lines represent individual mice per group. n=7 for PMX205, n=6 for vehicle.
- **(B)** Tumour growth curves are shown for MC38 subcutaneous tumours treated with either vehicle or PMX205 treatment for 3 doses (on day 0, 1 and 2). n=7 for PMX205, n=6 for vehicle.
- **(C)** Tumour growth curves are shown for MC38 subcutaneous tumours treated with 3 x 4.45 Gy single dose (equivalent to 9 Gy assuming an α/β ratio of 5.06)(Suwinski et al., 2007) and either vehicle or PMX205 treatment for 3 doses flanking RT (on day 0, 1 and 2). n=7/group.
- **(D)** Tumour growth curves are shown for MC38 subcutaneous tumours treated with 9 Gy single dose RT and either vehicle or PMX205 treatment for 3 doses flanking RT (on day 0, 1 and 2). n=7 for PMX205, n=8 for vehicle.

Supplementary Figure 6: Targeting C5aR1 does not increase the % of CD8+ T-cells in the tumour following RT

- **(A)** Gating strategy for the analysis shown in Figures 6B-L.
- **(B)** Gating strategy for the analysis shown in Figures 6M and N (and Supplementary Figures 6C-F).
- **(C)** Graph shows CD11b+ cells (as a % of live cells) in tumours of mice receiving 0 or 9 Gy and either vehicle or PMX205 treatment following the same dosing scheme as shown in Figure 6A. Tumours were harvested 7 days after RT. Individual points represent individual mice per group. n=5/group.
- **(D)** Graph shows NK cells (as a % of live cells) in tumours of mice receiving 0 or 9 Gy and either vehicle or PMX205 treatment following the same dosing scheme as shown in Figure 6A. Tumours were harvested 7 days after RT. Individual points represent individual mice per group.n=5/group.
- **(E)** Graph shows M-MDSC-cells (as a % of live cells) in tumours of mice receiving 0 or 9 Gy and either vehicle or PMX205 treatment following the same dosing scheme as shown in Figure 6A. Tumours were harvested 7 days after RT. Individual points represent individual mice per group.n=5/group.
- **(F)**Graph shows PMN-MDSC cells (as a % of live cells) in tumours of mice receiving 0 or 9 Gy and either vehicle or PMX205 treatment following the same dosing scheme as shown in Figure 6A. Tumours were harvested 7 days after RT. Individual points represent individual mice per group.n=5/group.

Supplementary Figure 7: C5aR1 inhibition can improve radiotherapy in tumours with an immunosuppressive microenvironment

- **(A)** AKPT organoids were treated (in vitro) with either vehicle or PMX205 for 48 hours. Western blotting was carried out with the antibodies indicated. β-actin was used as the loading control. n=3.
- **(B)** Tumour growth curves for AKPT organoids grown subcutaneously and treated with either 0 or 9 Gy and either vehicle or PMX205 flanking RT. Individual points = individual mice per group. n=7/group.
- **(C)** Tumour growth curves for AKPT organoids grown subcutaneously in athymic nude mice and treated with either 0 or 9 Gy and either vehicle or PMX205 flanking RT. Individual points = individual mice per group. n=7/group (n=6 for 0 Gy vehicle).

Supplementary Tables

Supplementary Table 1: List of GSEA significant pathways used to plot the data shown in Figure 1E; with corresponding columns denoting which ones are annotated as Complement and Immune System pathways.

Supplementary Table 2: Raw data used in the generation of the heatmap shown in Figure 2A, see also methods section.

Supplementary Table 3: Clinical characteristics of rectal adenocarcinoma patients undergoing long-course chemoradiotherapy.

Supplementary Table 4: Pathways differentially expressed by RPPA following treatment with PMX205.

Supplementary Table 5: Genes differentially expressed by RPPA following treatment with PMX205.

Supplementary Table 6: Exact p-values for correlations shown in Figure 3F.

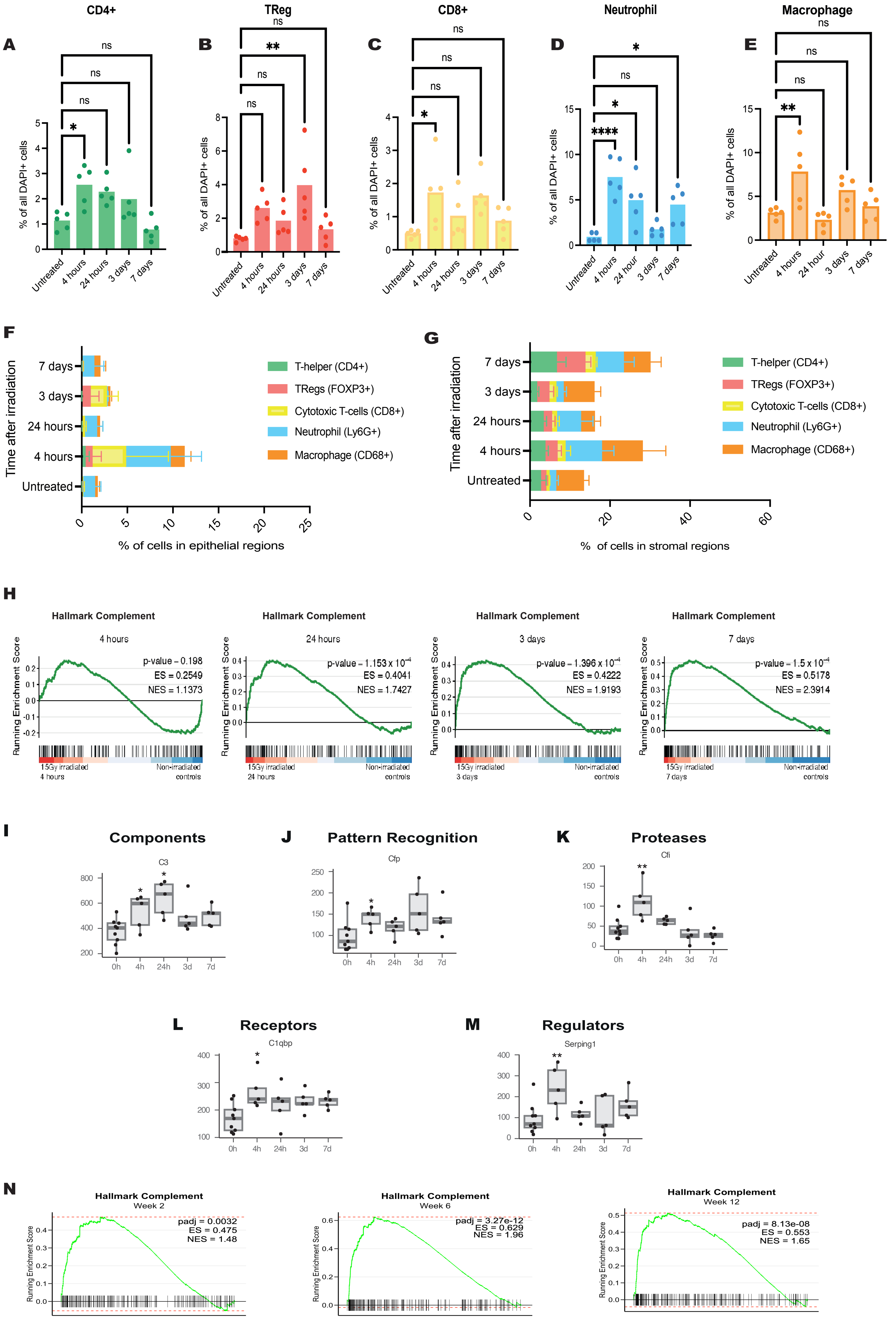

A
CD4+
B
TReg
C
CD8+
D
Neutrophil
E
Macrophage
% of all DAPI+ cells
Untreated
4 hours
24 hours
3 days
7 days
F
Time after irradiation
% of cells in epithelial regions
T-helper (CD4+)
TRegs (FOXP3+)
Cytotoxic T-cells (CD8+)
Neutrophil (Ly6G+)
Macrophage (CD68+)
G
% of cells in stromal regions
H
Hallmark Complement
4 hours
p-value = 0.198
ES = 0.2549
NES = 1.1373
24 hours
p-value = 1.153 x 10^-4
ES = 0.4041
NES = 1.7427
3 days
p-value = 1.396 x 10^-4
ES = 0.4222
NES = 1.9193
7 days
p-value = 1.5 x 10^-4
ES = 0.5178
NES = 2.3914
Running Enrichment Score
15Gy irradiated
Non-irradiated controls
I
Components
C3
J
Pattern Recognition
Cfp
K
Proteases
Cfi
L
Receptors
C1qbp
M
Regulators
Serping1
N
Hallmark Complement
Week 2
padj = 0.0032
ES = 0.475
NES = 1.48
Week 6
padj = 3.27e-12
ES = 0.629
NES = 1.96
Week 12
padj = 8.13e-08
ES = 0.553
NES = 1.65

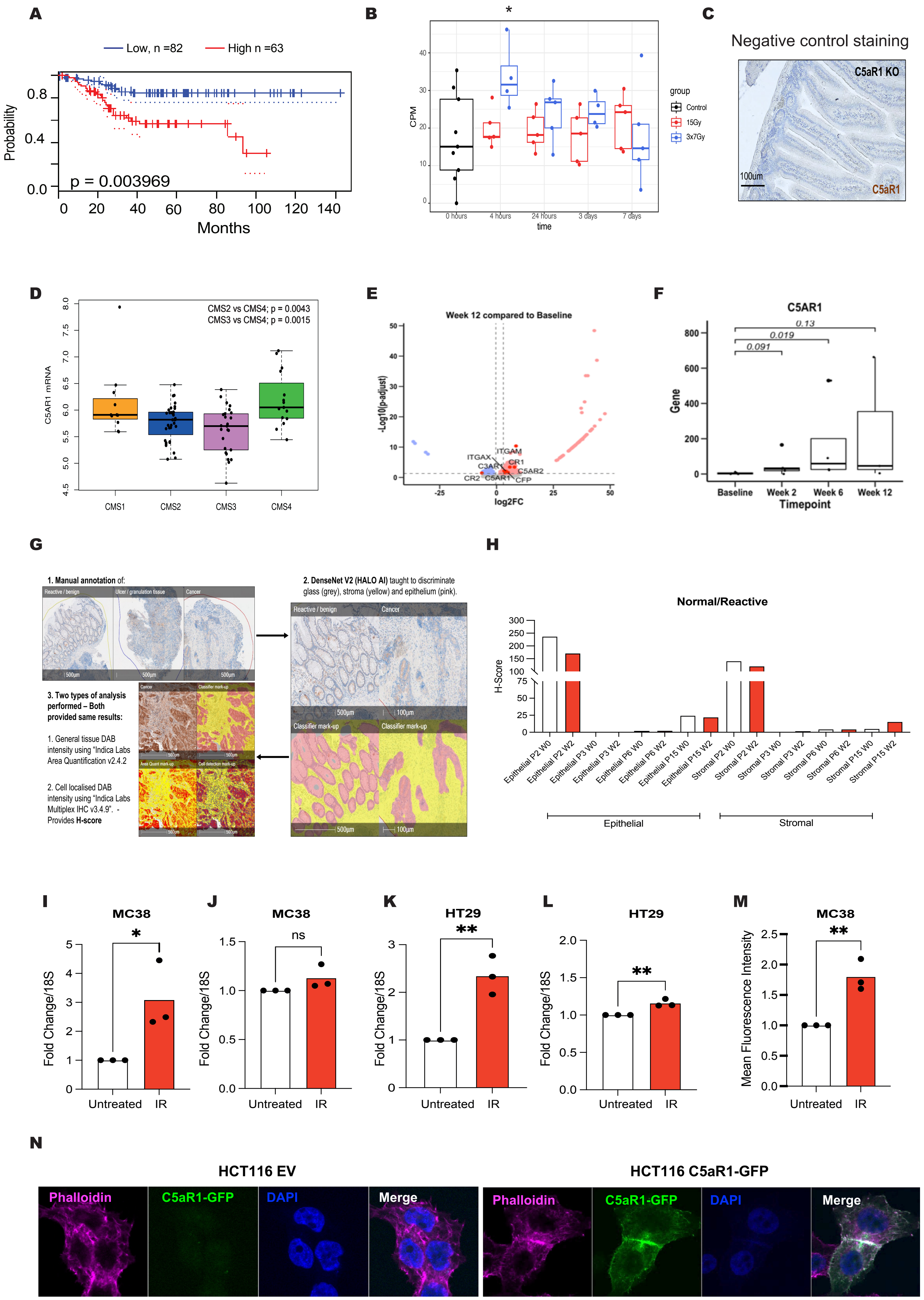
A
Low, n =82
High n =63
Probability
p = 0.003969
Months
B
CPM
group
Control
15Gy
3x7Gy
0 hours
4 hours
24 hours
3 days
7 days
time
C
Negative control staining
C5aR1 KO
100um
C5aR1
D
C5AR1 mRNA
CMS2 vs CMS4; p = 0.0043
CMS3 vs CMS4; p = 0.0015
CMS1
CMS2
CMS3
CMS4
E
Week 12 compared to Baseline
-Log10(p-adjust)
log2FC
ITGAM
ITGAX
C3AR1
CR1
C5AR2
CR2
C5AR1
CFP
F
C5AR1
Gene
0.13
0.019
0.091
Baseline
Week 2
Week 6
Week 12
Timepoint
G
1. Manual annotation of:
Reactive / benign
Ulcer / granulation tissue
Cancer
2. DenseNet V2 (HALO AI) taught to discriminate glass (grey), stroma (yellow) and epithelium (pink).
3. Two types of analysis performed – Both provided same results:
1. General tissue DAB intensity using "Indica Labs Area Quantification v2.4.2
2. Cell localised DAB intensity using "Indica Labs Multiplex IHC v3.4.9". - Provides H-score
Classifier mark-up
Area Quant mark-up
Cell detection mark-up
H
Normal/Reactive
H-Score
Epithelial P2 W0
Epithelial P2 W2
Epithelial P3 W0
Epithelial P3 W2
Epithelial P6 W0
Epithelial P6 W2
Epithelial P15 W0
Epithelial P15 W2
Stromal P2 W0
Stromal P2 W2
Stromal P3 W0
Stromal P3 W2
Stromal P6 W0
Stromal P6 W2
Stromal P15 W0
Stromal P15 W2
Epithelial
Stromal
I
MC38
Fold Change/18S
Untreated
IR
J
MC38
ns
K
HT29
L
HT29
M
MC38
Mean Fluorescence Intensity
N
HCT116 EV
HCT116 C5aR1-GFP
Phalloidin
C5aR1-GFP
DAPI
Merge

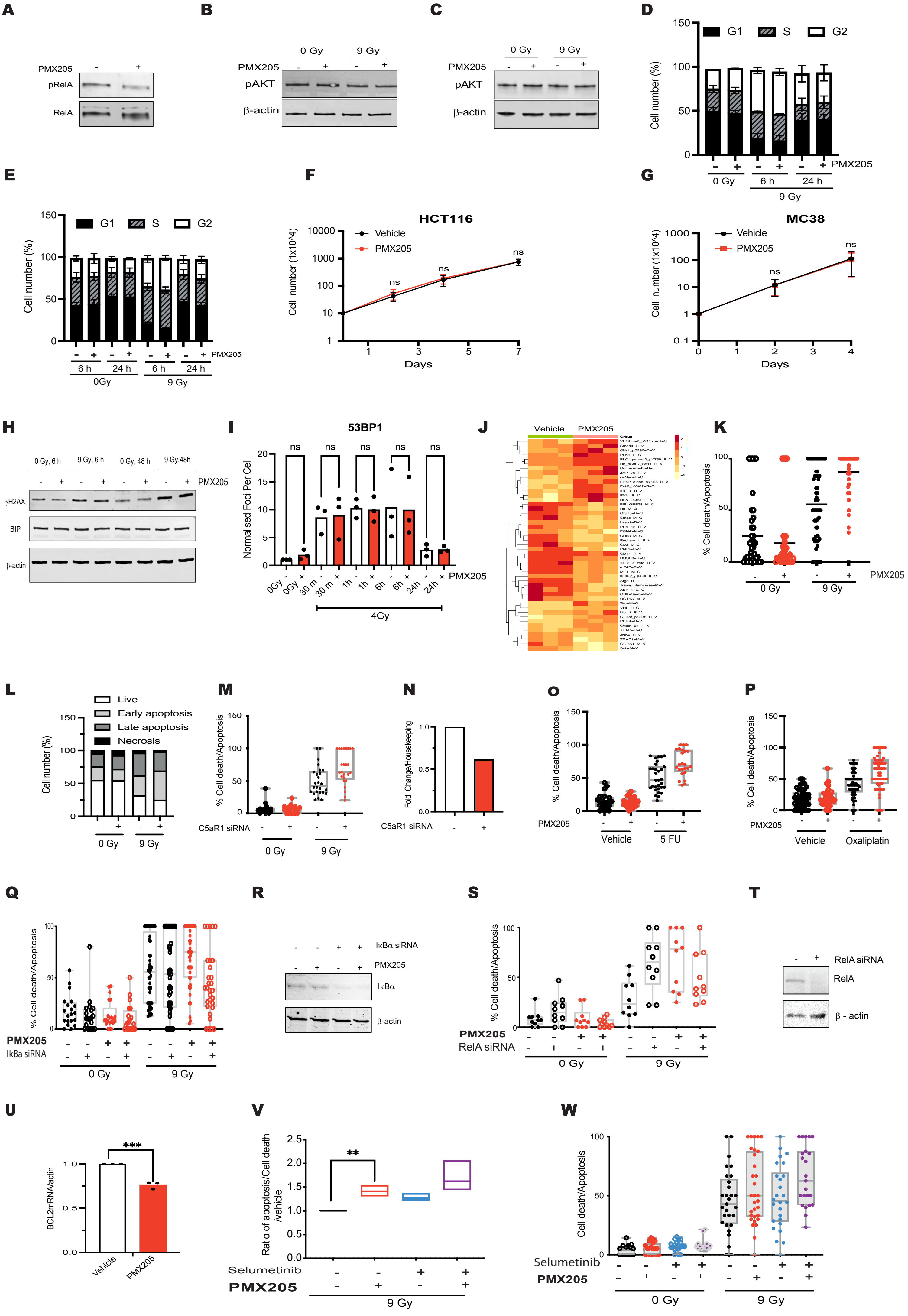

A
PMX205 - +
pRelA
RelA
B
0 Gy 9 Gy
PMX205 - + - +
pAKT
β-actin
C
0 Gy 9 Gy
PMX205 - + - +
pAKT
β-actin
D
G1 S G2
Cell number (%)
150 100 50 0
- + - + - + PMX205
0 Gy 6 h 24 h
9 Gy
E
G1 S G2
Cell number (%)
150 100 50 0
- + - + - + - + PMX205
6 h 24 h 6 h 24 h
0Gy 9 Gy
F
HCT116
Vehicle
PMX205
Cell number (1x10^4)
10000 1000 100 10 1
ns ns ns
1 3 5 7
Days
G
MC38
Vehicle
PMX205
Cell number (1x10^4)
1000 100 10 1 0.1
ns ns
0 1 2 3 4
Days
H
0 Gy, 6 h 9 Gy, 6 h 0 Gy, 48 h 9 Gy,48h
- + - + - + - + PMX205
γH2AX
BIP
β-actin
I
53BP1
Normalised Foci Per Cell
20 15 10 5 0
ns ns ns ns ns
0Gy - 0Gy + 30 m - 30 m + 1h - 1h + 6h - 6h + 24h - 24h + PMX205
4Gy
J
Vehicle PMX205
K
% Cell death/Apoptosis
100 50 0
- + - + PMX205
0 Gy 9 Gy
L
Live
Early apoptosis
Late apoptosis
Necrosis
Cell number (%)
150 100 50 0
- + - +
0 Gy 9 Gy
M
% Cell death/Apoptosis
150 100 50 0
C5aR1 siRNA - + - +
0 Gy 9 Gy
N
Fold Change/Housekeeping
1.0 0.5 0.0
C5aR1 siRNA - +
O
% Cell death/Apoptosis
150 100 50 0
PMX205 - + - +
Vehicle 5-FU
P
% Cell death/Apoptosis
150 100 50 0
PMX205 - + - +
Vehicle Oxaliplatin
Q
% Cell death/Apoptosis
100 50 0
PMX205 - - + + - - + +
IkBa siRNA - + - + - + - +
0 Gy 9 Gy
R
- - + + IκBα siRNA
- + - + PMX205
IκBα
β-actin
S
% Cell death/Apoptosis
100 50 0
PMX205 - - + + - - + +
RelA siRNA - + - + - + - +
0 Gy 9 Gy
T
- + RelA siRNA
RelA
β - actin
U
***
BCL2mRNA/actin
1.0 0.5 0.0
Vehicle PMX205
V
**
Ratio of apoptosis/Cell death /vehicle
2.5 2.0 1.5 1.0 0.5 0.0
Selumetinib - - + +
PMX205 - + - +
9 Gy
W
Cell death/Apoptosis
100 50 0
Selumetinib - - + + - - + +
PMX205 - + - + - + - +
0 Gy 9 Gy

A

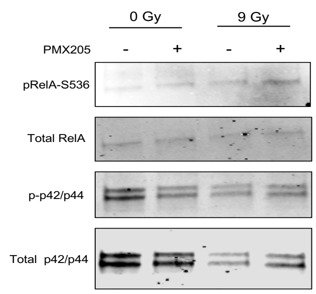

B

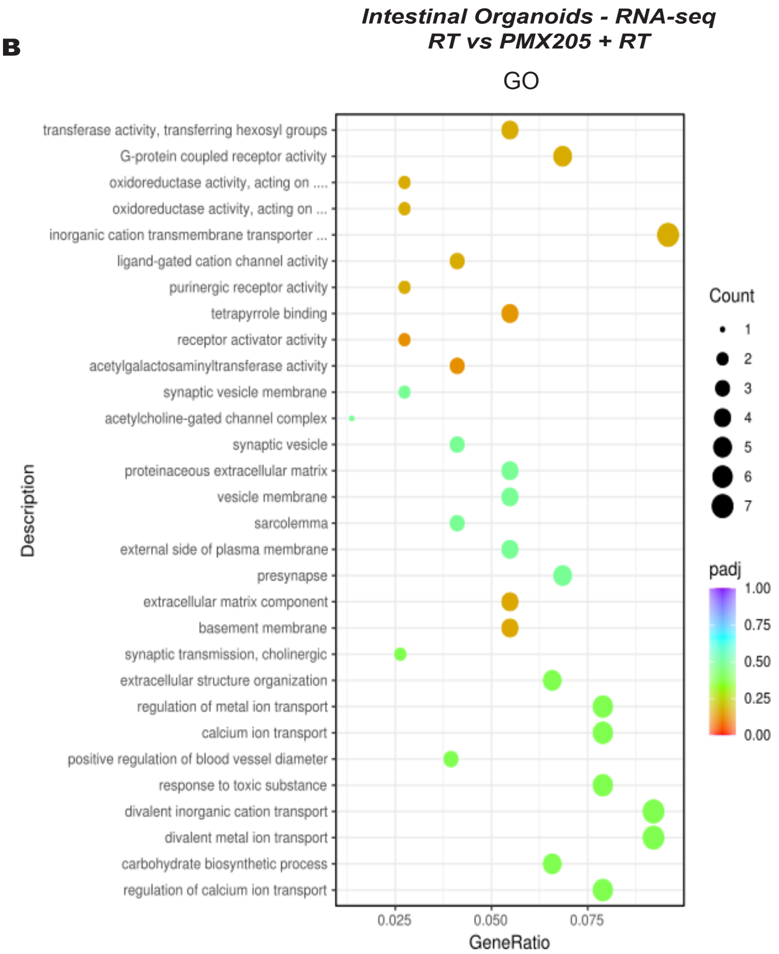

C

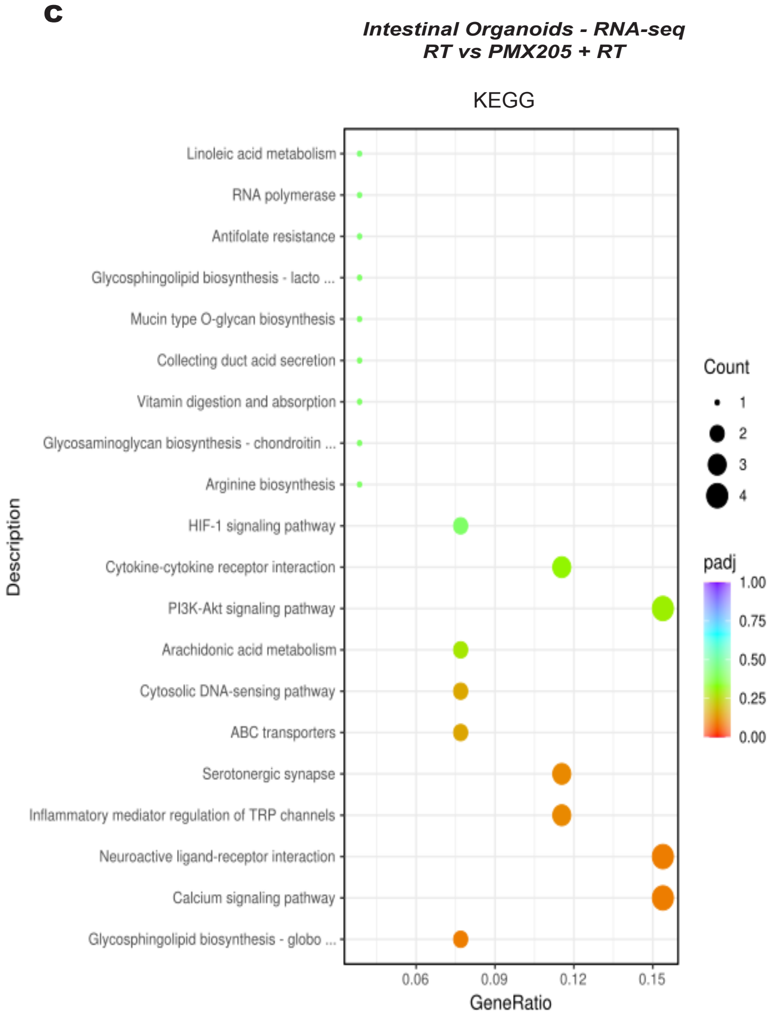

D

Intestinal Organoids - RNA-seq
RT vs PMX205 + RT

Gene Name	Log2FoldChange	pvalue	padj
ASC	0.460742397	0.462567929	0.999048582
Bax	-0.074392938	0.664320749	0.999048582
BCL-XL	-0.077364081	0.468877536	0.999048582
BCL2	0.044741456	0.800000596	0.999048582
Bim	0.223643113	0.16199464	0.990208917
B7-H1	-0.057357814	0.86283745	0.999048582
BNIP3	0.223520801	0.02292787	0.584499123

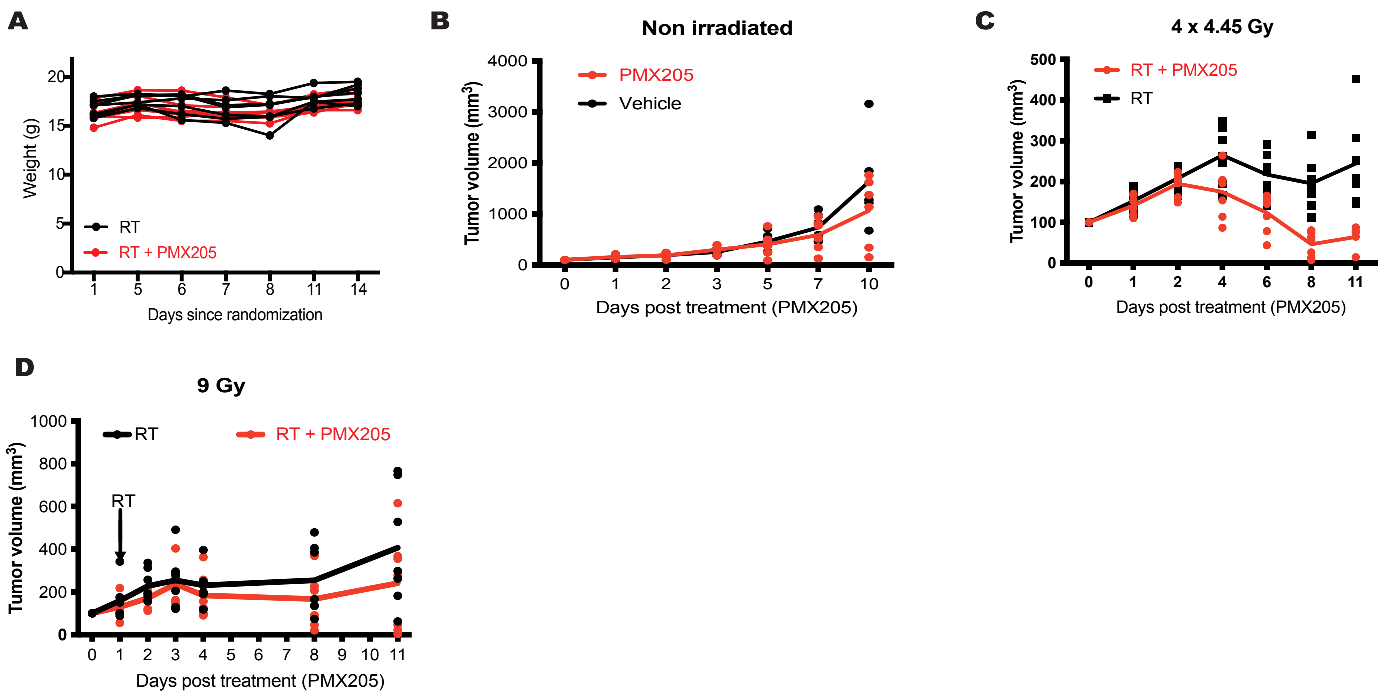
A
Weight (g)
RT
RT + PMX205
Days since randomization
B
Non irradiated
Tumor volume (mm³)
PMX205
Vehicle
Days post treatment (PMX205)
C
4 x 4.45 Gy
Tumor volume (mm³)
RT + PMX205
RT
Days post treatment (PMX205)
D
9 Gy
Tumor volume (mm³)
RT
RT + PMX205
RT
Days post treatment (PMX205)

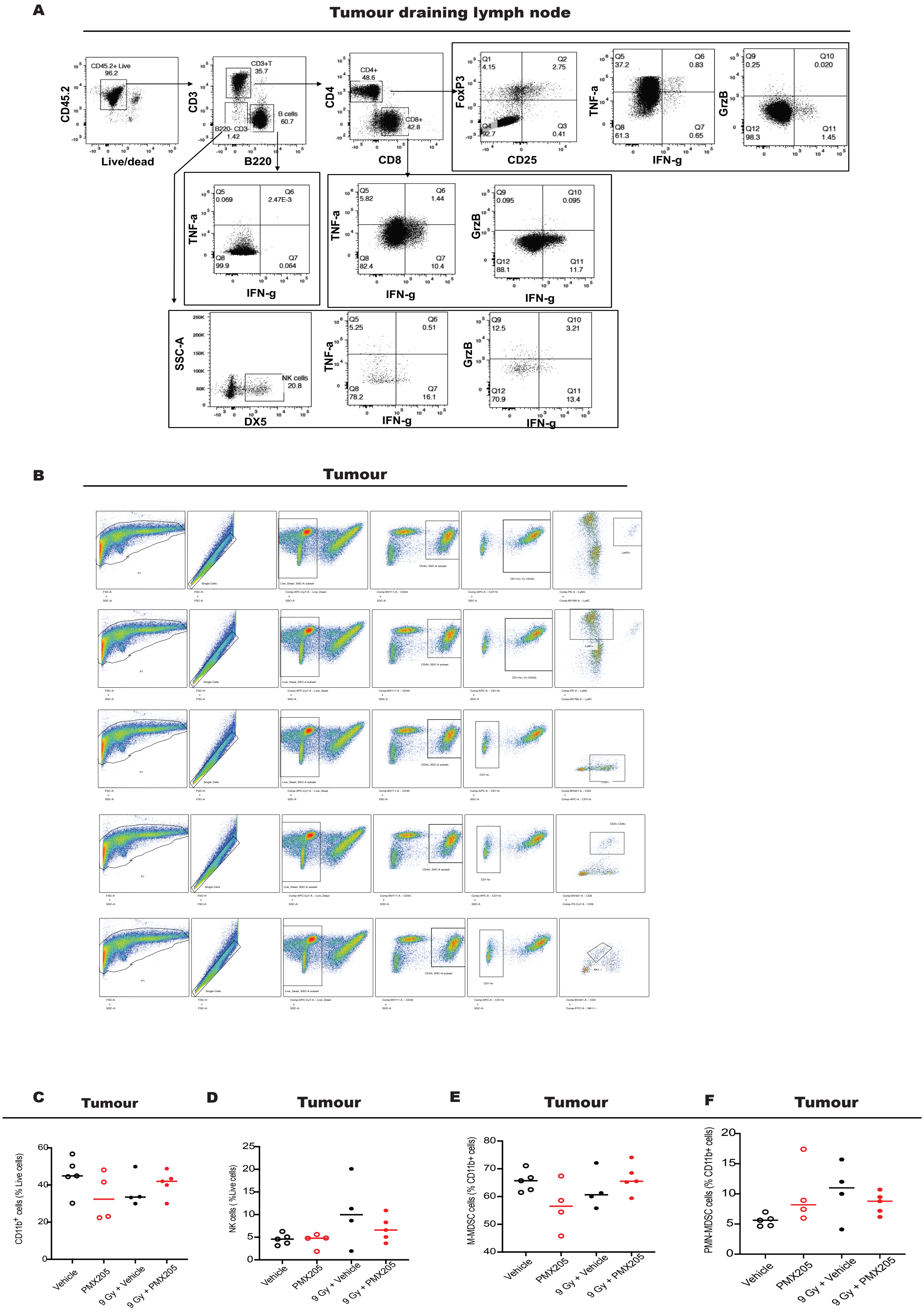
A
Tumour draining lymph node
CD45.2+ Live
96.2
CD45.2
Live/dead
CD3+T
35.7
B cells
60.7
B220- CD3-
1.42
CD3
B220
CD4+
48.6
CD8+
42.8
CD4
CD8
Q1
4.15
Q2
2.75
Q3
0.41
Q4
92.7
FoxP3
CD25
Q5
37.2
Q6
0.83
Q7
0.65
Q8
61.3
TNF-a
IFN-g
Q9
0.25
Q10
0.020
Q11
1.45
Q12
98.3
GrzB
IFN-g
Q5
0.069
Q6
2.47E-3
Q7
0.064
Q8
99.9
TNF-a
IFN-g
Q5
5.82
Q6
1.44
Q7
10.4
Q8
82.4
TNF-a
IFN-g
Q9
0.095
Q10
0.095
Q11
11.7
Q12
88.1
GrzB
IFN-g
SSC-A
DX5
NK cells
20.8
Q5
5.25
Q6
0.51
Q7
16.1
Q8
78.2
TNF-a
IFN-g
Q9
12.5
Q10
3.21
Q11
13.4
Q12
70.9
GrzB
IFN-g
B
Tumour
C
Tumour
CD11b+ cells (% Live cells)
Vehicle
PMX205
9 Gy + Vehicle
9 Gy + PMX205
D
Tumour
NK cells (%Live cells)
Vehicle
PMX205
9 Gy + Vehicle
9 Gy + PMX205
E
Tumour
M-MDSC cells (% CD11b+ cells)
Vehicle
PMX205
9 Gy + Vehicle
9 Gy + PMX205
F
Tumour
PMN-MDSC cells (% CD11b+ cells)
Vehicle
PMX205
9 Gy + Vehicle
9 Gy + PMX205

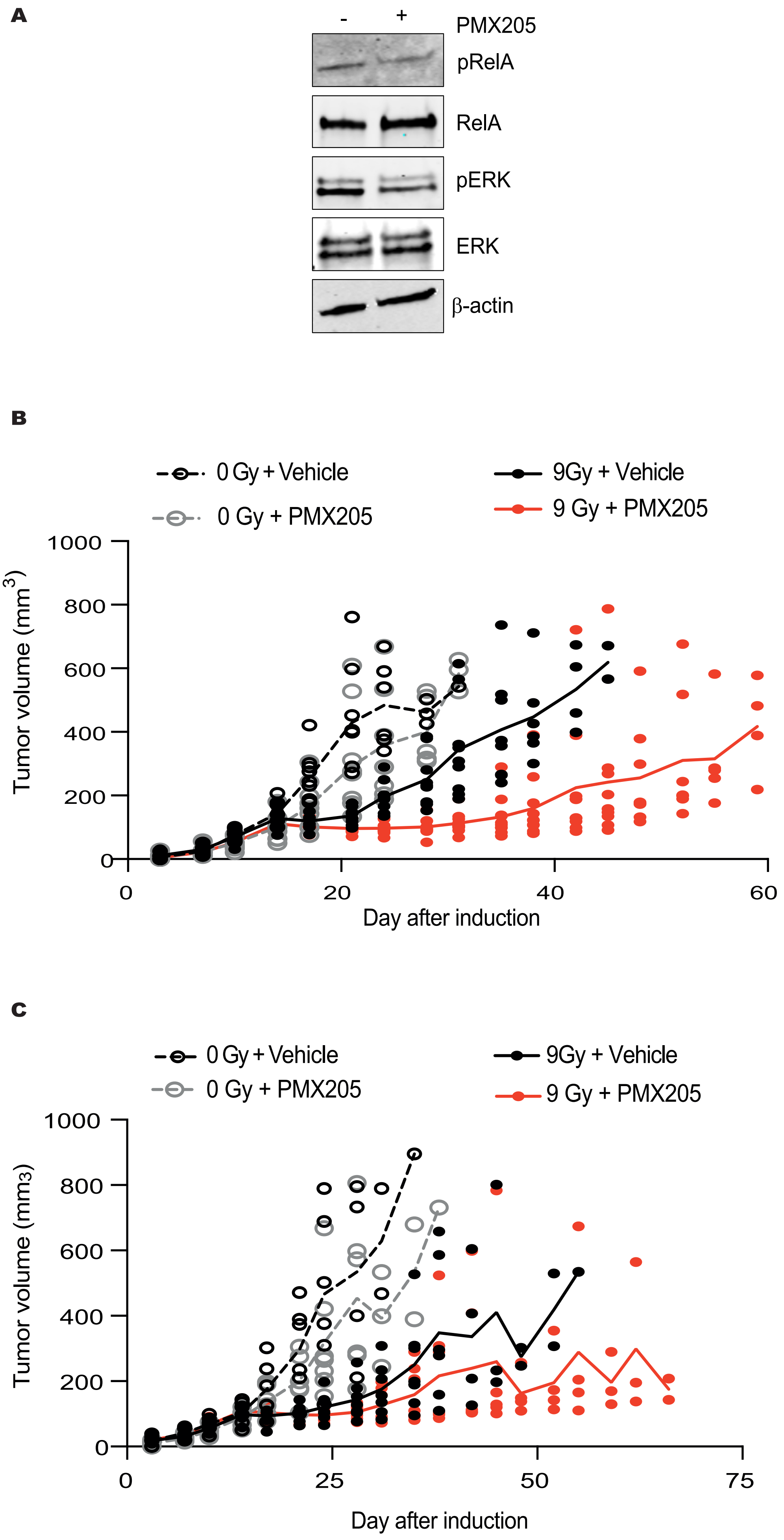
A
-
+
PMX205
pRelA
RelA
pERK
ERK
β-actin
B
0 Gy + Vehicle
0 Gy + PMX205
9Gy + Vehicle
9 Gy + PMX205
Tumor volume (mm3)
1000
800
600
400
200
0
0
20
40
60
Day after induction
C
0 Gy + Vehicle
0 Gy + PMX205
9Gy + Vehicle
9 Gy + PMX205
Tumor volume (mm3)
1000
800
600
400
200
0
0
25
50
75
Day after induction

Full unedited gel for Figure 3A

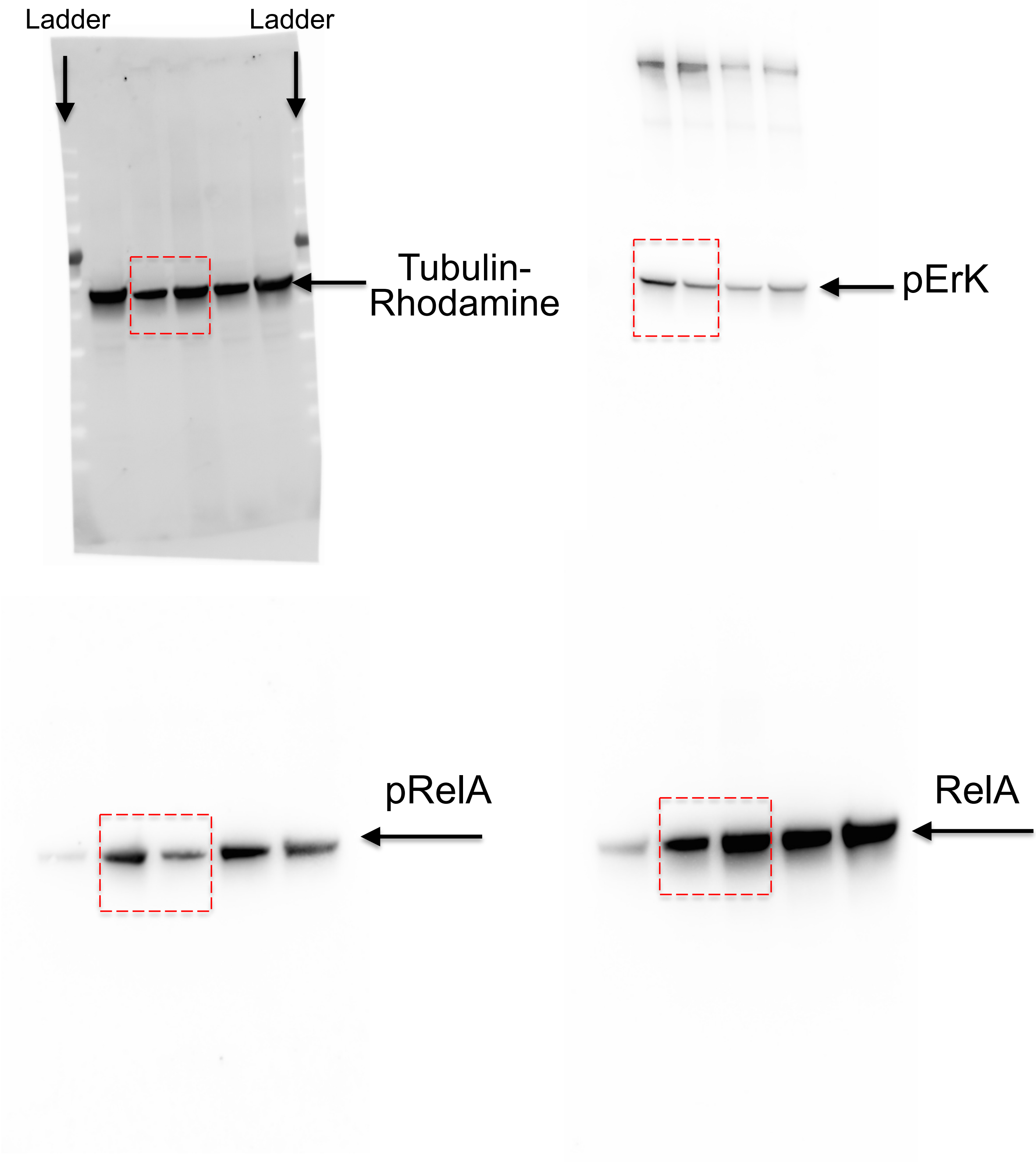

Full unedited gel for Figure 3B

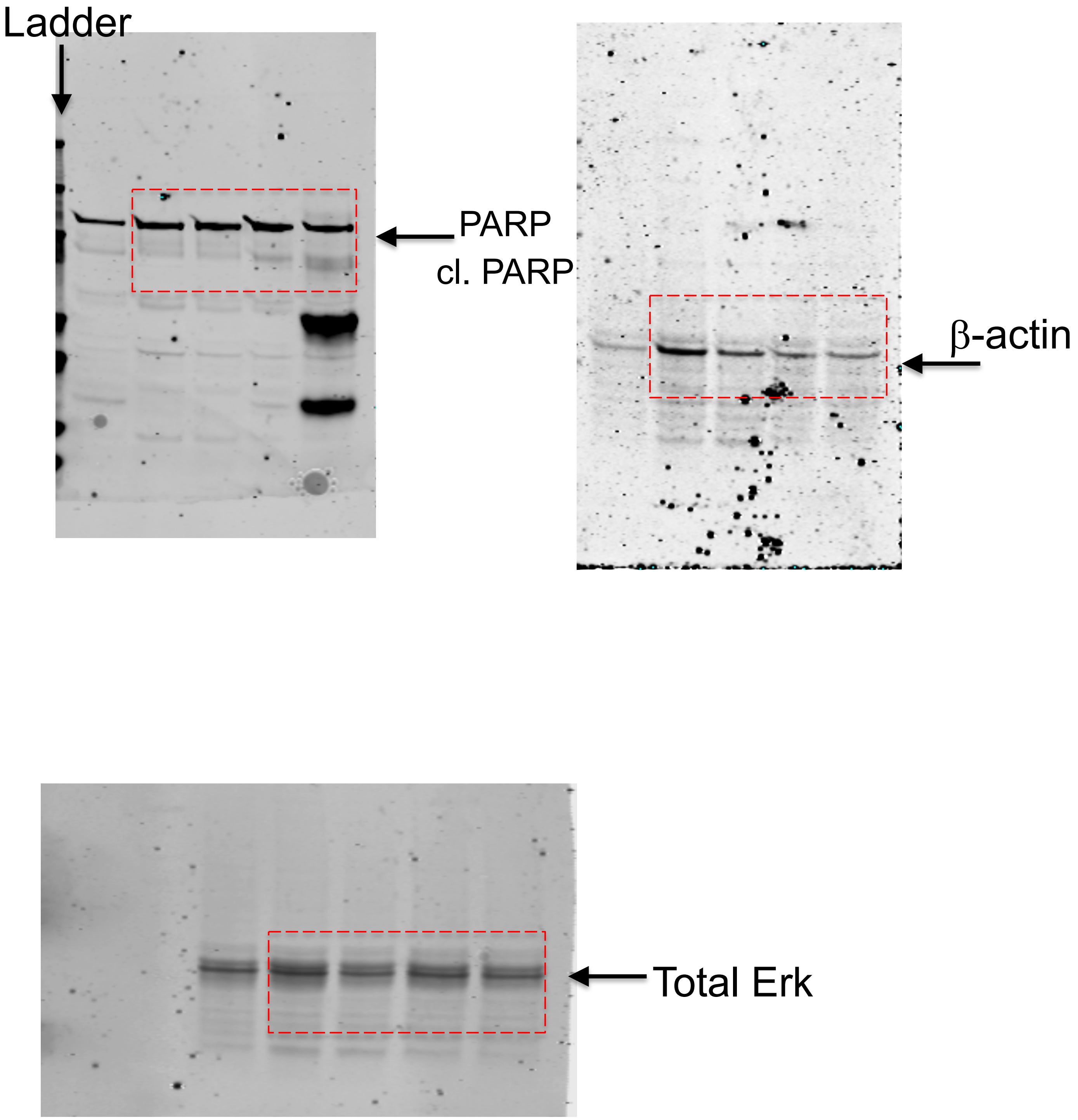

Full unedited gel for Supplementary Figure 3A

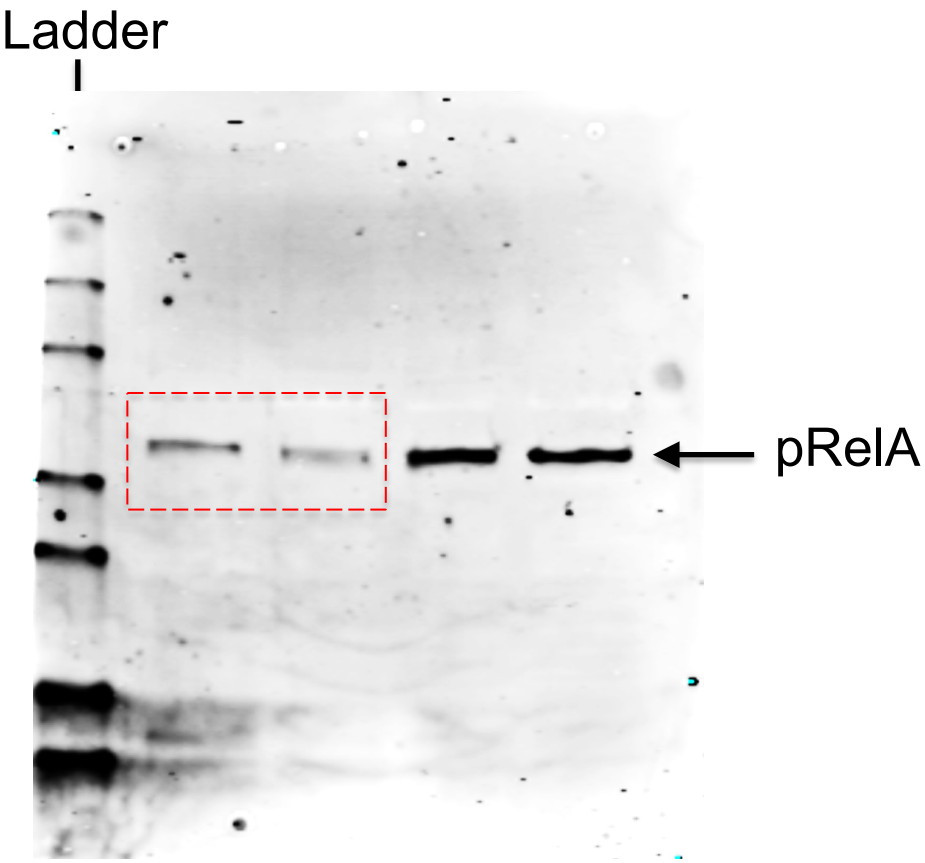

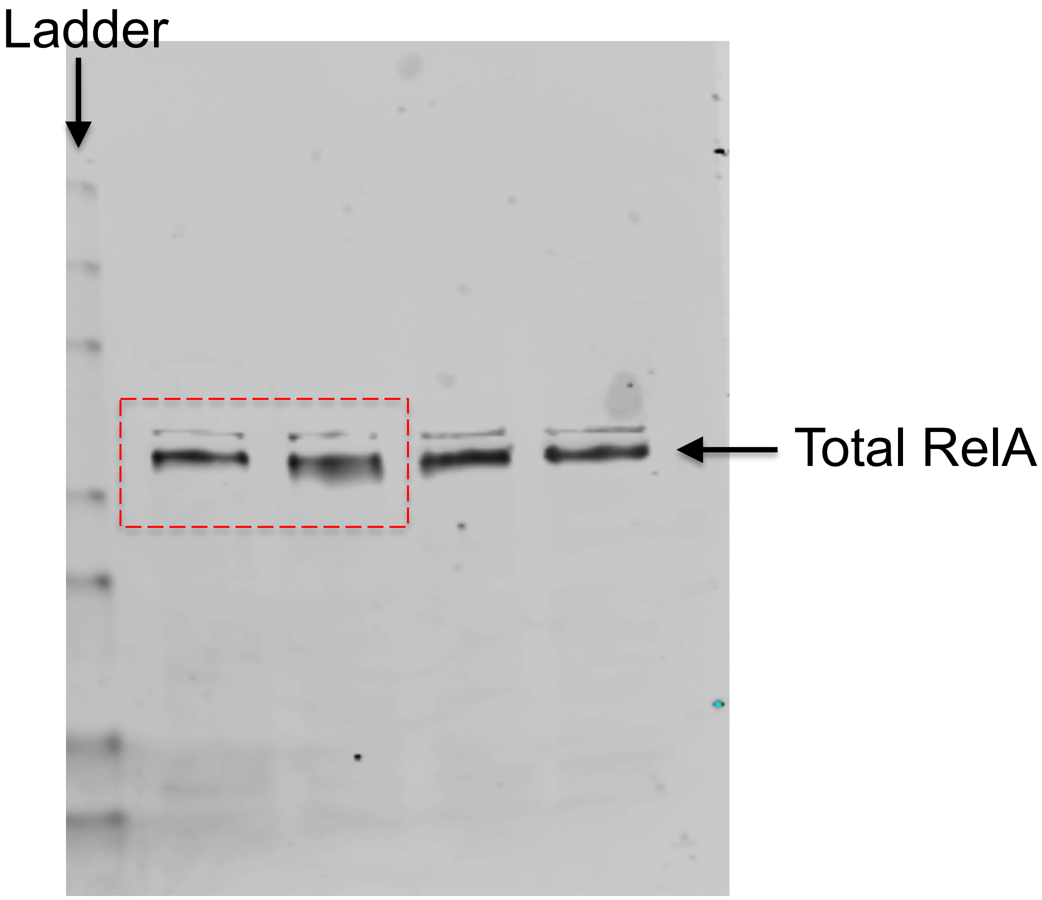

Full unedited gel for Supplementary Figure 3B

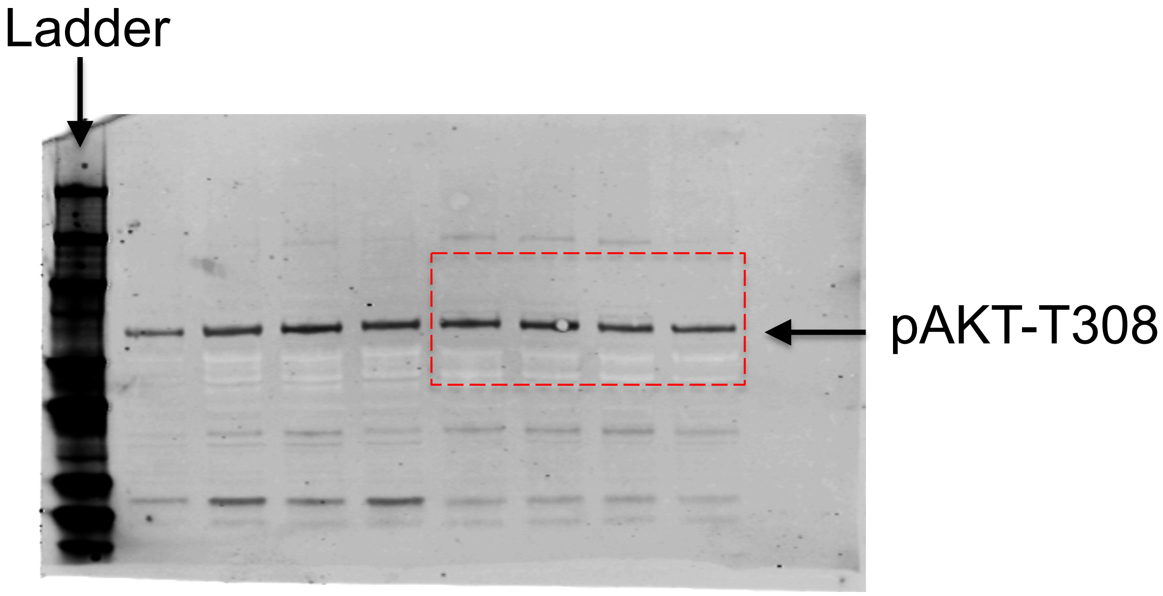

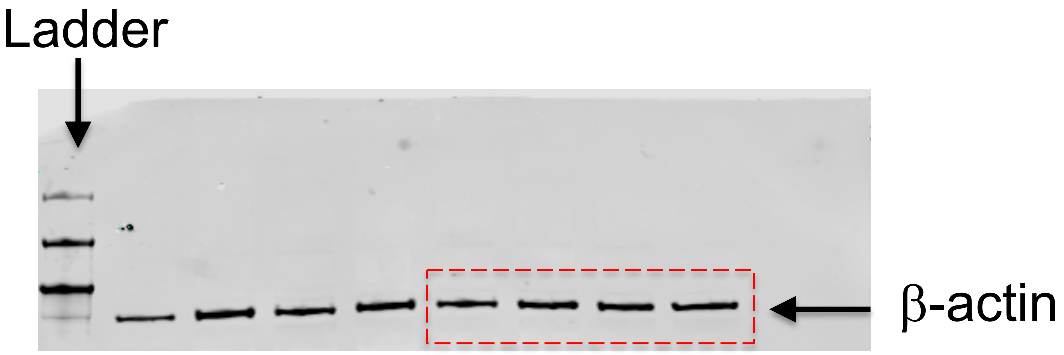

Full unedited gel for Supplementary Figure 3C

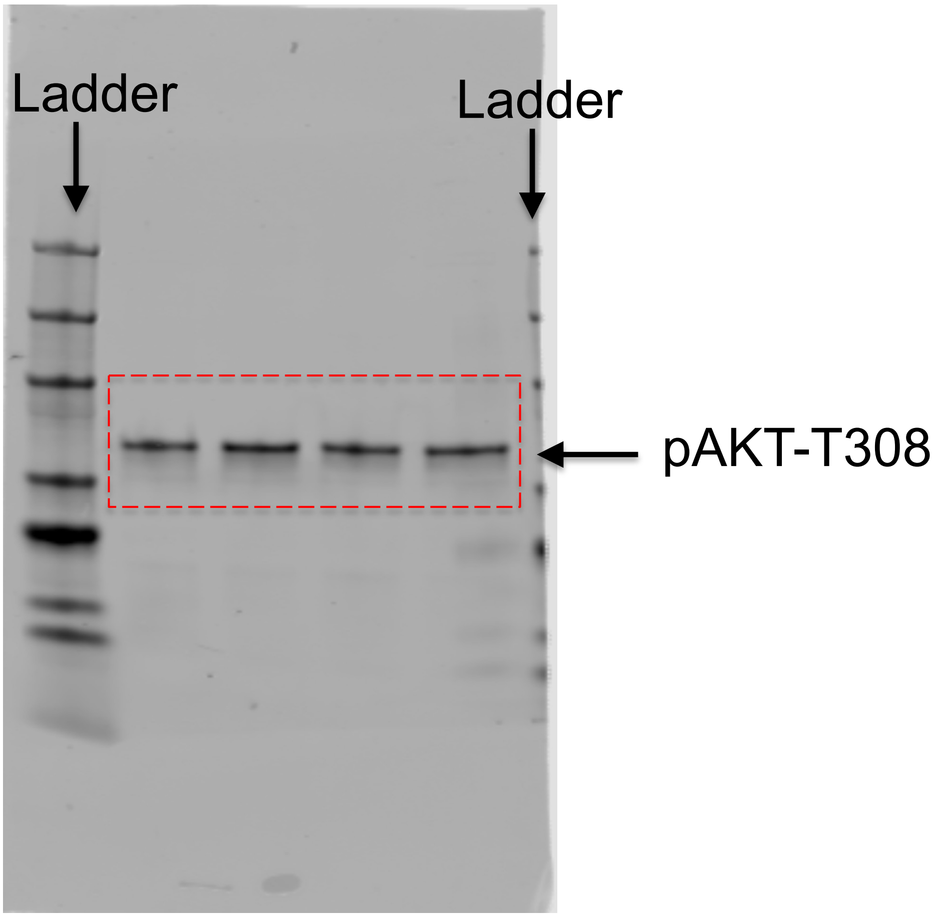

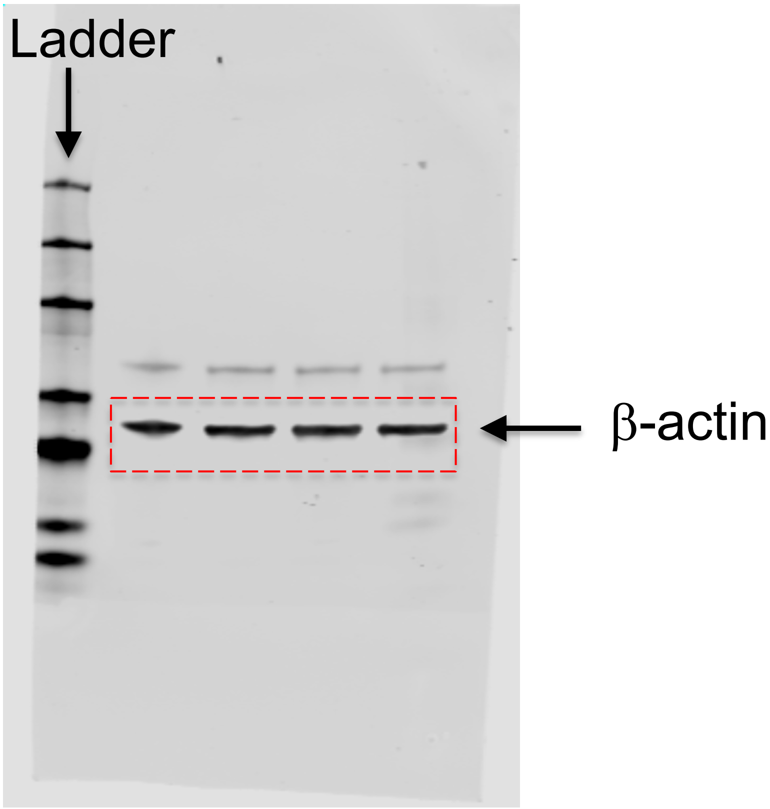

Full unedited gel for Supplementary Figure 3H

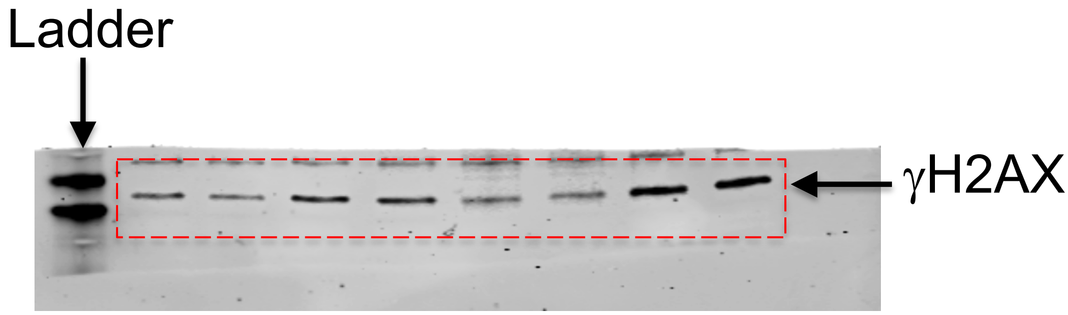

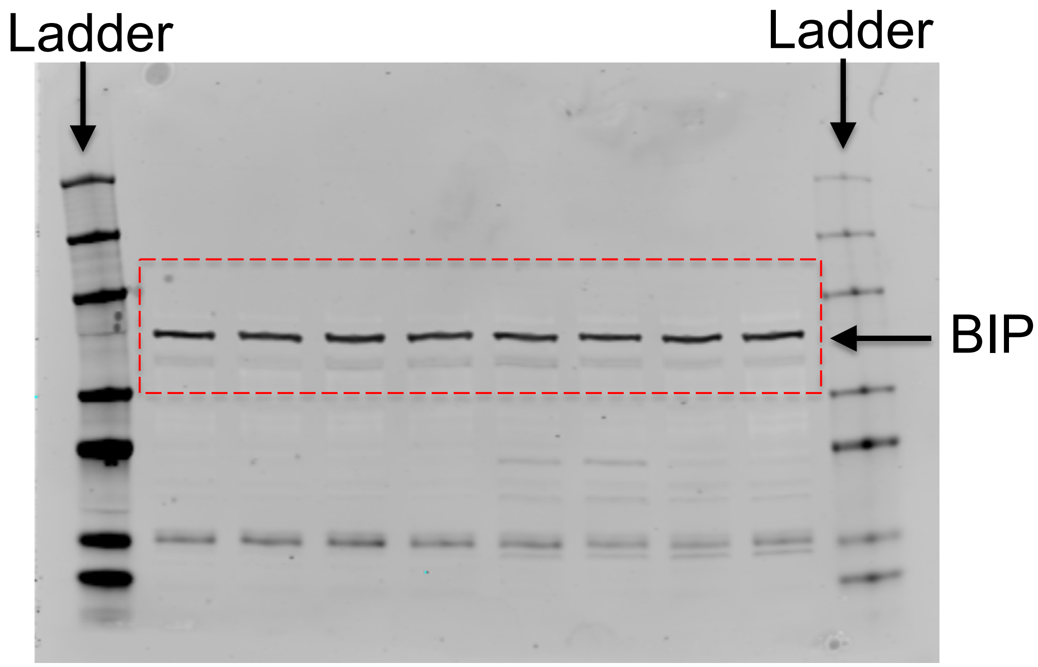

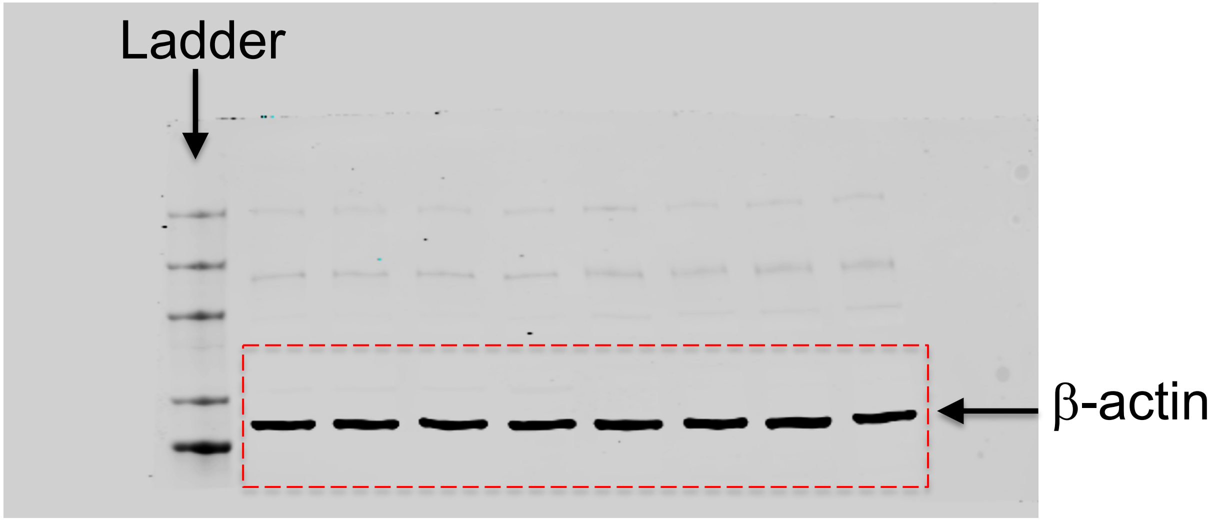

Full unedited gel for Supplementary Figure 3R

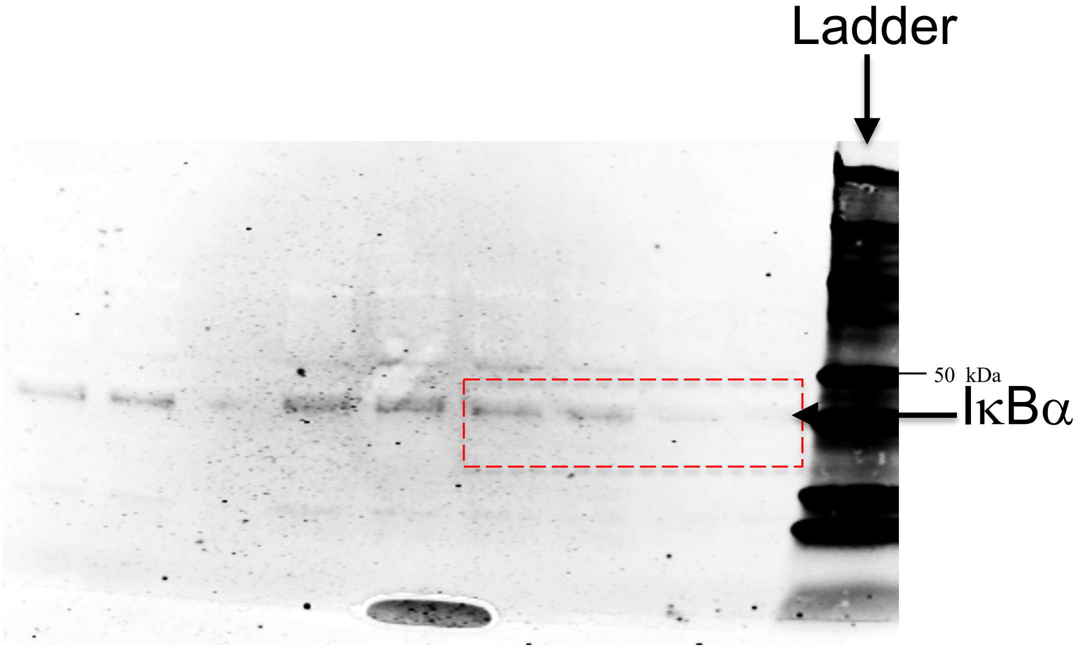

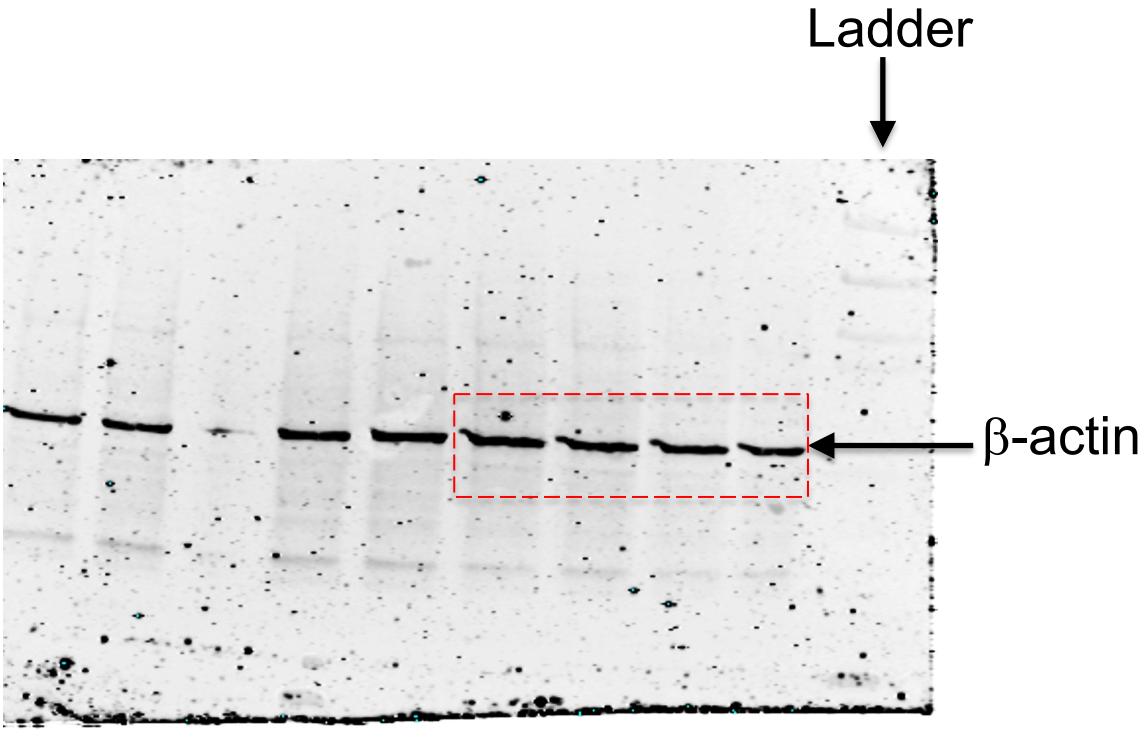

Full unedited gel for Supplementary Figure 3T

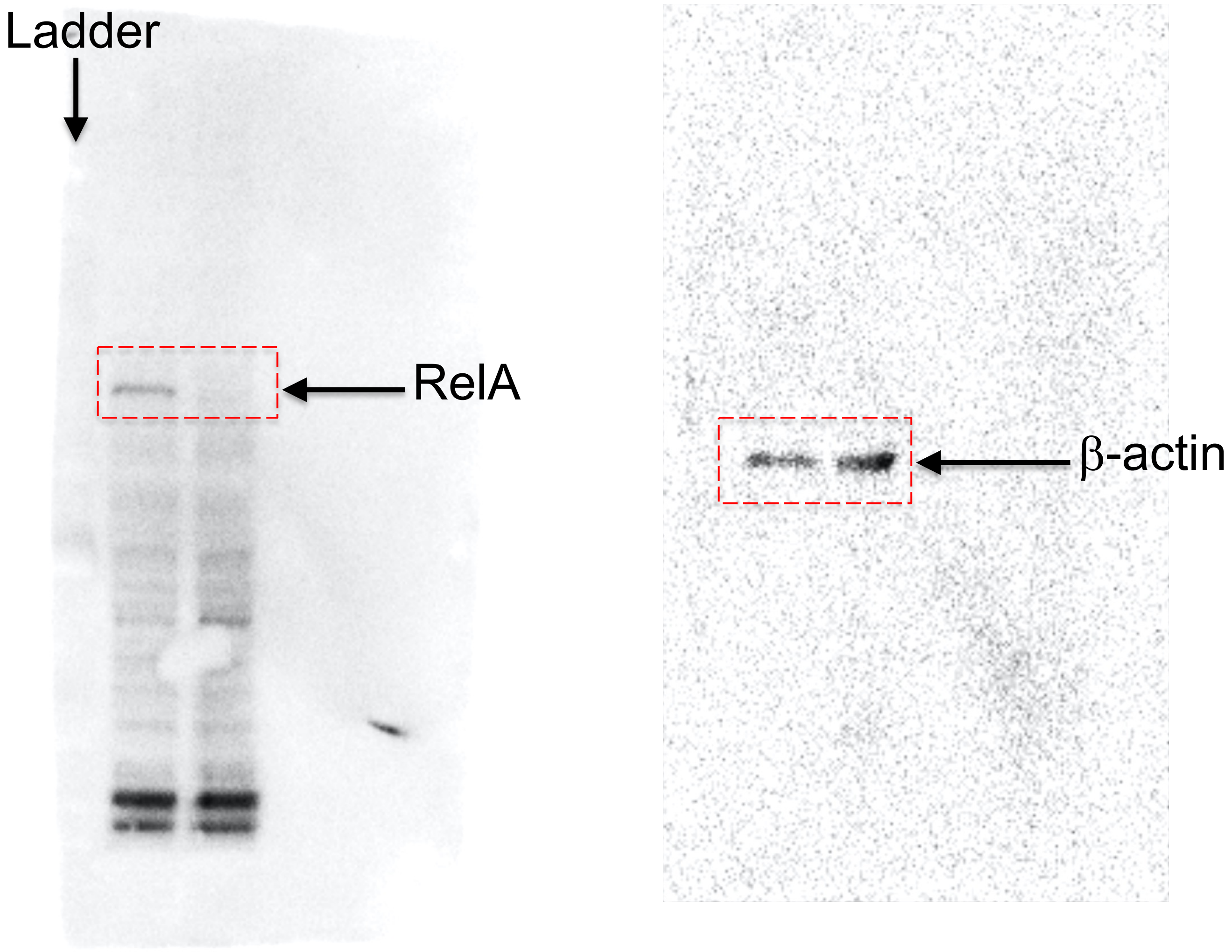

Full unedited gel for Supplementary Figure 4A

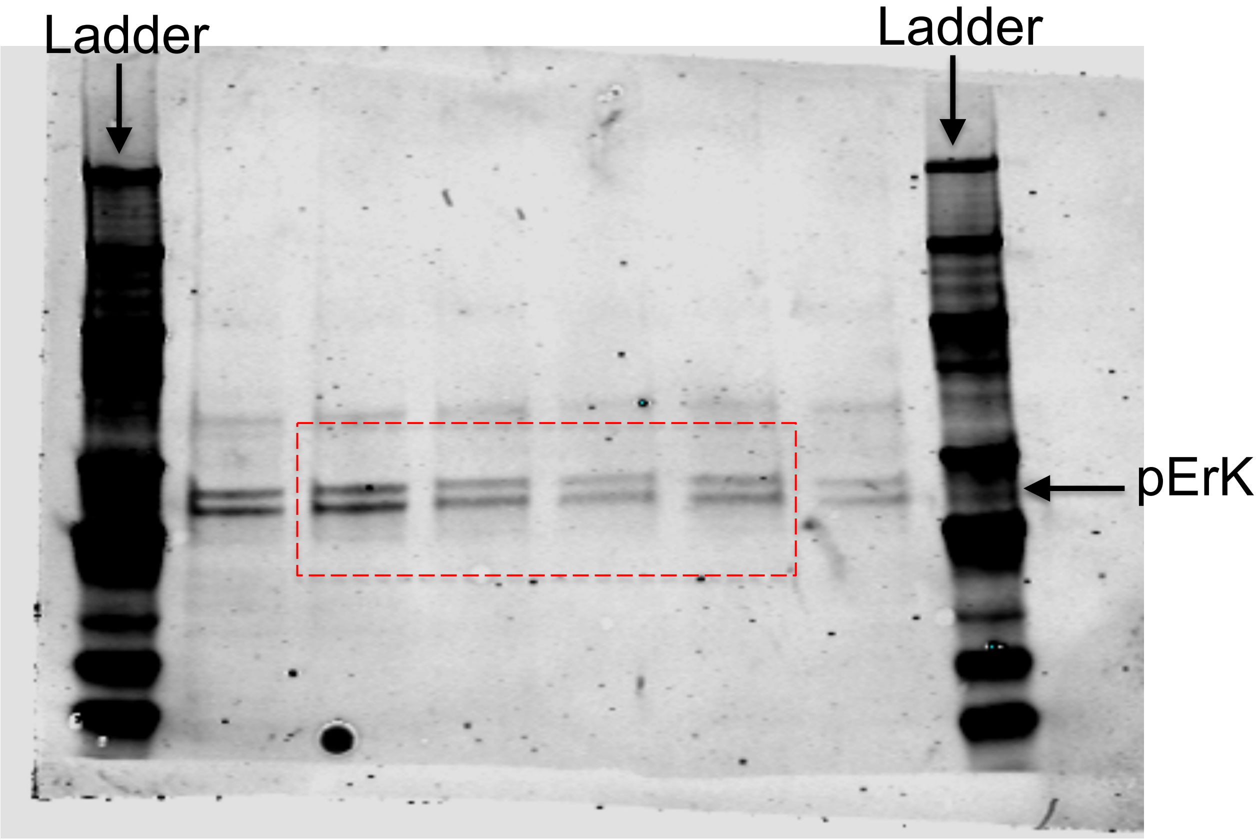

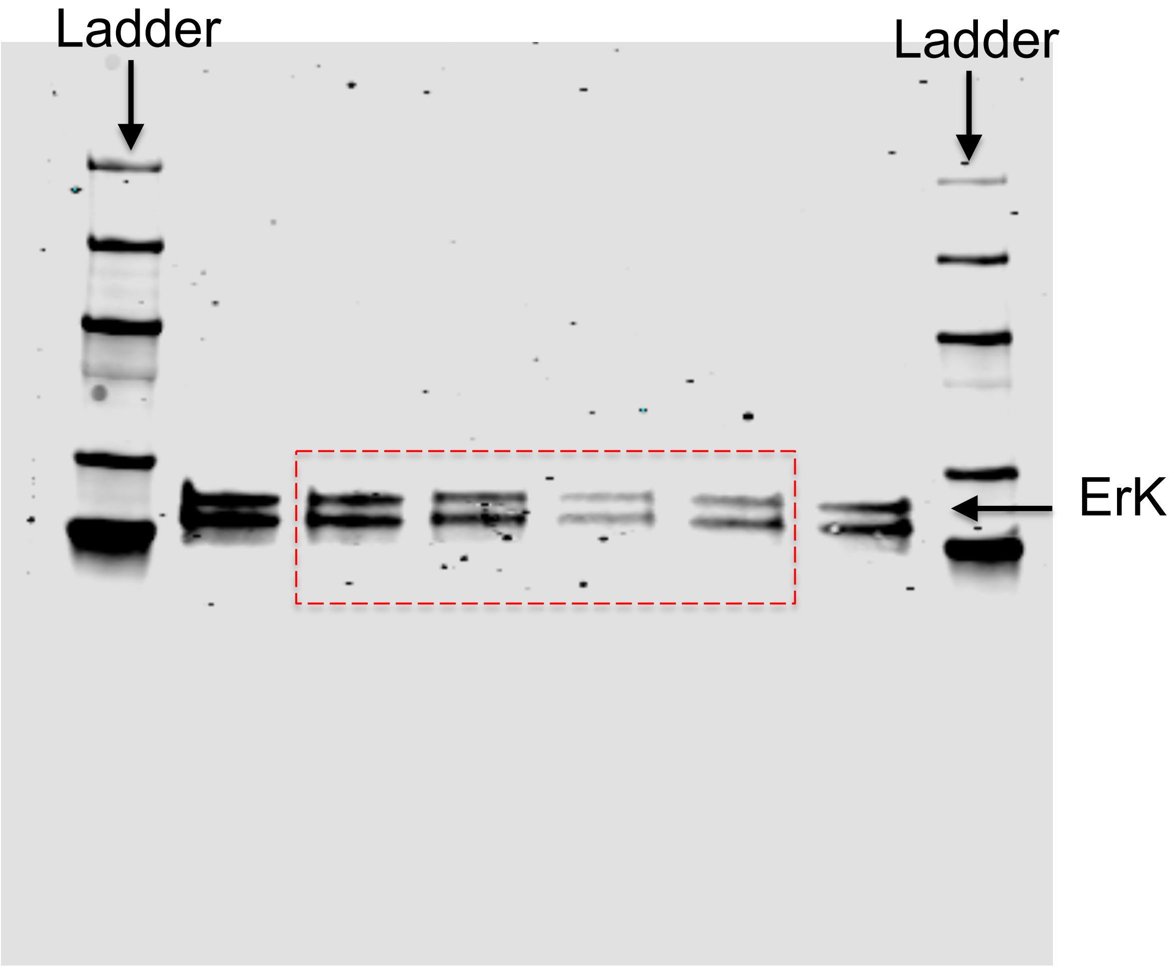

Full unedited gel for Supplementary Figure 4A

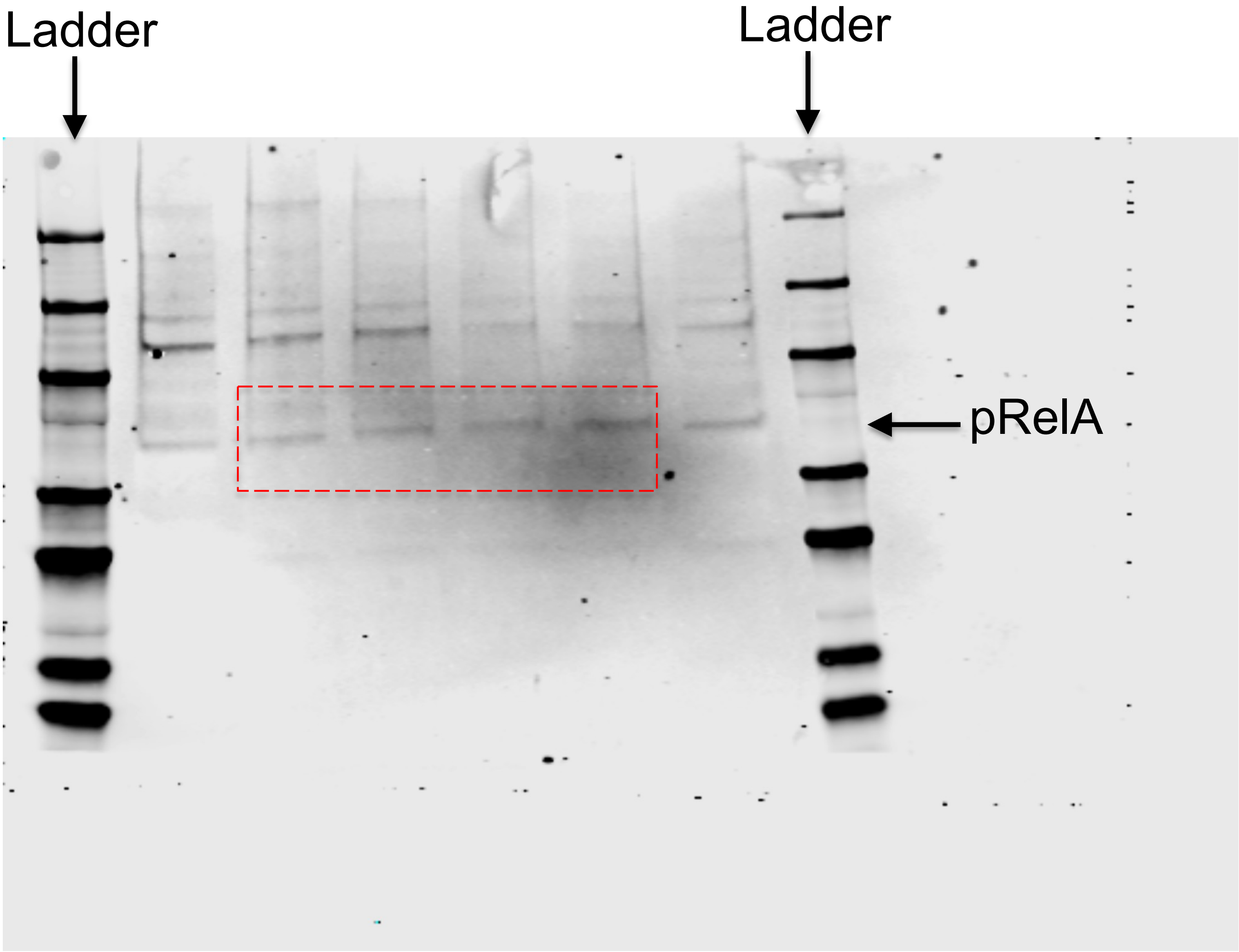

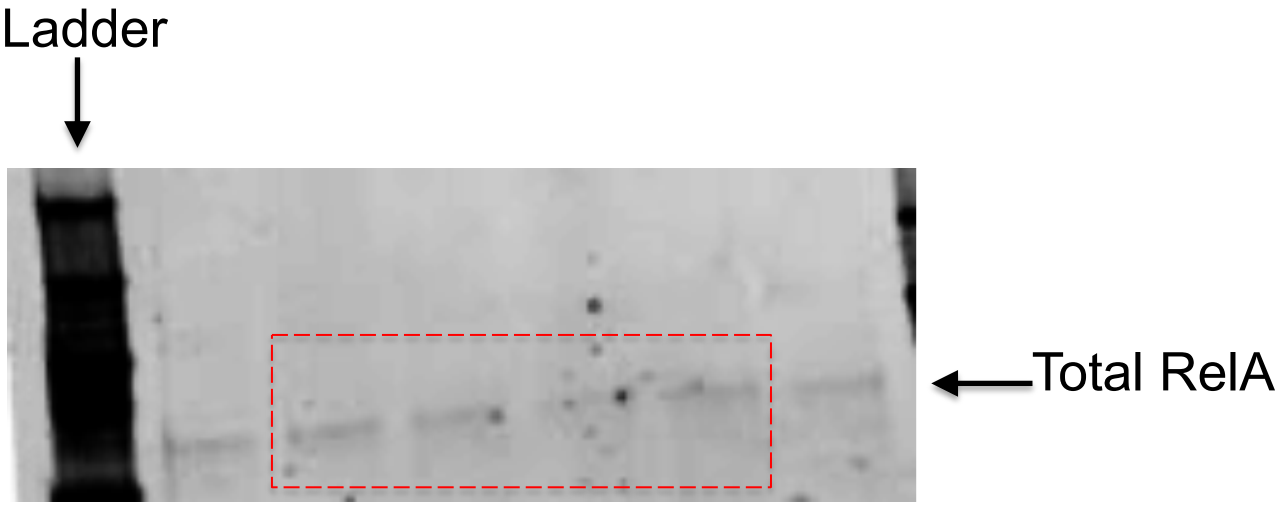

Full unedited gel for Supplementary Figure 7A

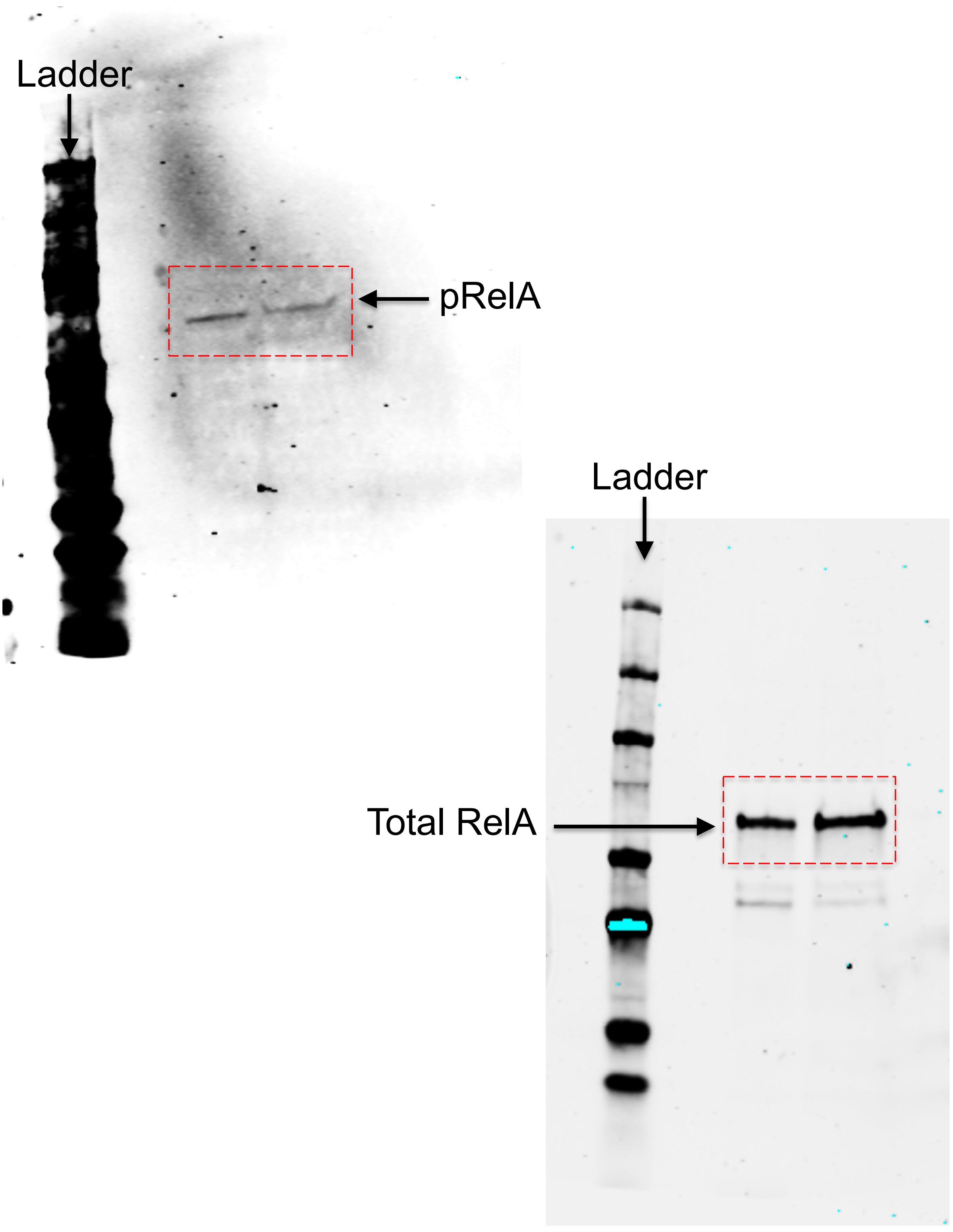

Full unedited gel for Supplementary Figure 7A Continued

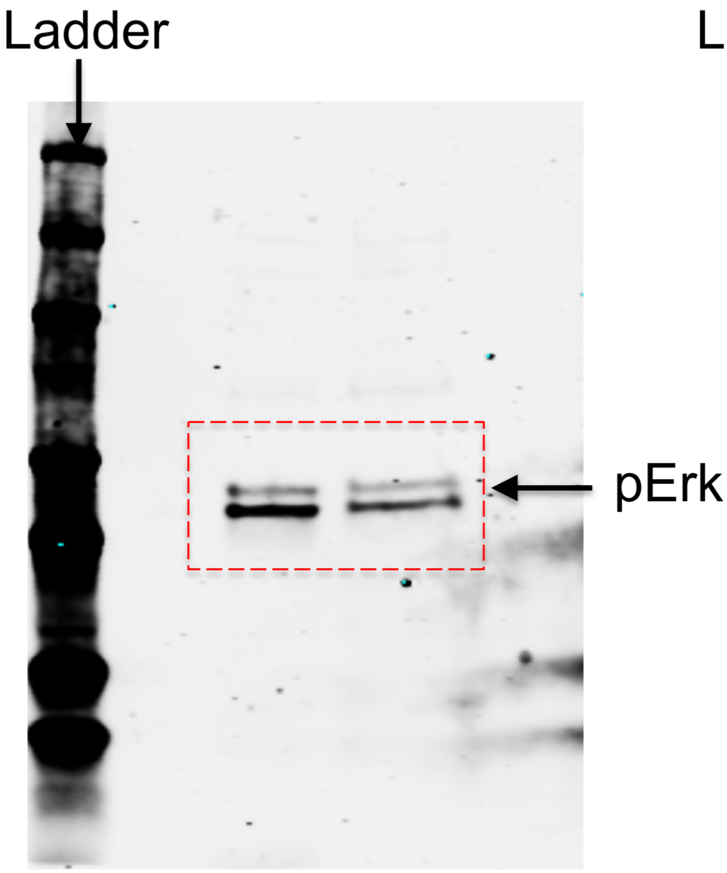

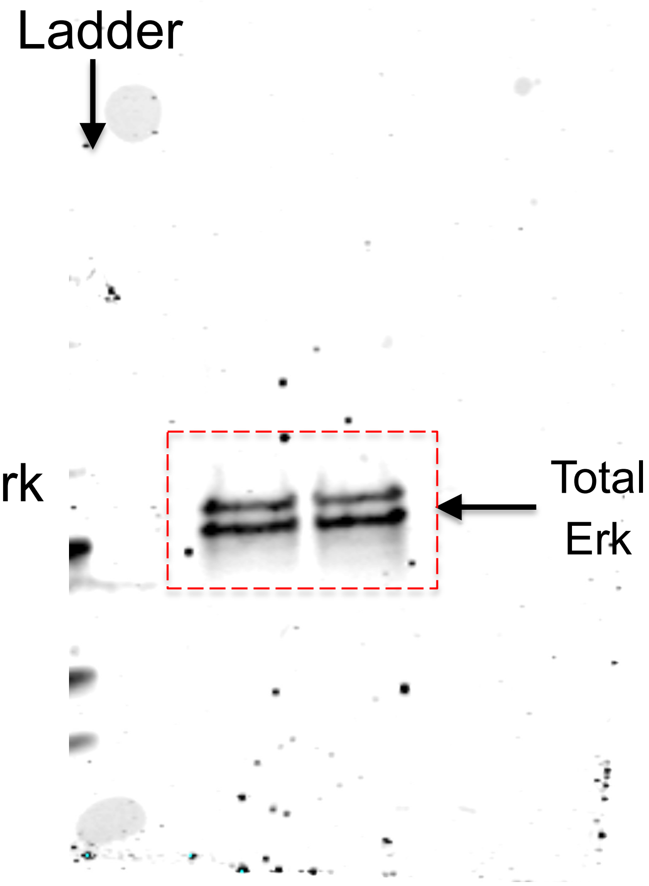

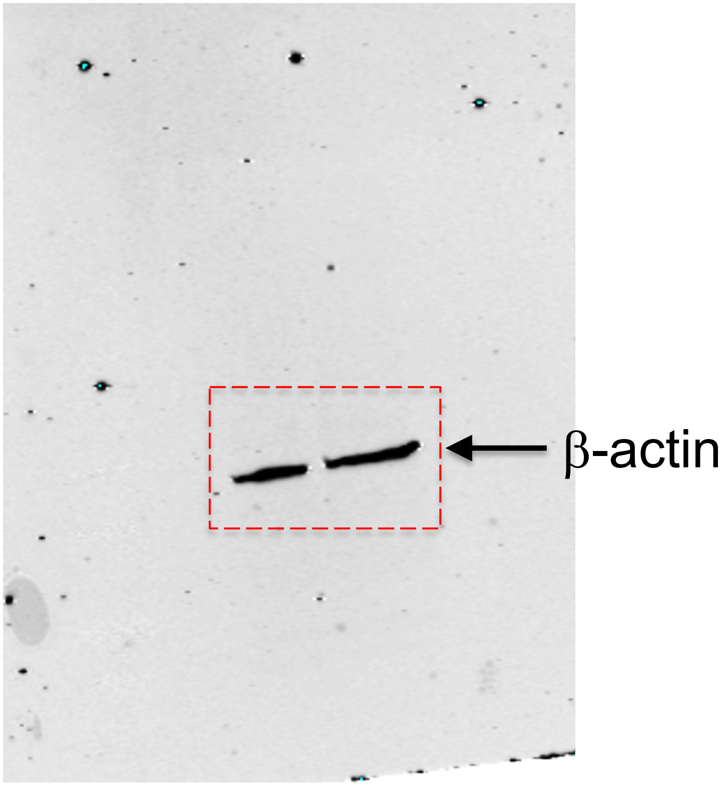

Supplement: Supplemental data [file jci-133-168277-s059.pdf]
